# *F2RL3* Methylation in the Peripheral Blood as a Potential Marker for the Detection of Coronary Heart Disease: A Case-Control Study

**DOI:** 10.3389/fgene.2022.833923

**Published:** 2022-03-24

**Authors:** Xiaojing Zhao, Liya Zhu, Qiming Yin, Zhenguo Xu, Qian Jia, Rongxi Yang, Kunlun He

**Affiliations:** ^1^ Military Translational Medicine Lab, Medical Innovation Research Division, Chinese PLA General Hospital, Beijing, China; ^2^ Beijing Key Laboratory of Chronic Heart Failure Precision Medicine, Medical Innovation Research Division, Chinese PLA General Hospital, Beijing, China; ^3^ Department of Epidemiology and Biostatistics, School of Public Health, Nanjing Medical University, Nanjing, China; ^4^ The First Medical Center, Chinese PLA General Hospital, Beijing, China; ^5^ The Medical School of Chinese PLA, Beijing, China

**Keywords:** epigenomics, coronary heart disease, DNA methylation, coagulation factor II receptor-like 3 gene, biomarker, blood

## Abstract

**Background and Aims:** Previous work has shown the association between blood-based methylation of coagulation factor II receptor-like 3 gene (*F2RL3*) and cardiovascular mortality in Caucasians. However, the diagnostic value of *F2RL3* methylation for CHD is still unknown. The aim of our study was to evaluate the association between blood-based *F2RL3* methylation and the risk of CHD in the Chinese population.

**Methods:** The methylation level of *F2RL3* was quantified by mass spectrometry in a case-control study with 180 CHD cases and 184 controls. The association between *F2RL3* methylation intensity and CHD was assessed by logistic regression models, controlling confounding factors.

**Results:** The hypomethylation in F2RL3_A amplicon was significantly associated with CHD (odds ratio (ORs) per -10% methylation: 1.22–1.42, *p* < 0.035 for six out of seven CpG loci). Specifically, this significant association was observed in elderly CHD patients (≥60 years), myocardial infarction (MI) patients, heart failure patients and the patients with minor to medium cardiac function impairment (NYHA Ⅰ&Ⅱ CHD cases) (ORs per -10% methylation: 1.35–1.58, 1.32–2.00, 1.29–1.43, 1.25–1.44; *p* < 0.024, 0.033, 0.035, 0.025, respectively). However, F2RL3_B CpG sites showed no or very weak association with CHD. The combination of F2RL3_A_CpG_1 and F2RL3_A_CpG_3 methylation levels could efficiently discriminate CHD, MI, heart failure, NYHA I&II CHD, and elderly CHD patients from controls (area under curve (AUC) = 0.75, 0.79, 0.75, 0.76, and 0.82, respectively).

**Conclusion:** We propose blood-based *F2RL3* methylation as a potential biomarker for CHD, especially for people with older age or with the status of MI. The combination of *F2RL3* methylation and conventional risk factors might be an approach to evaluate CHD at early stage.

## Introduction

Coronary heart disease (CHD) is the leading cause of morbidity and mortality worldwide, producing immense health and economic burdens globally (Shaya et al., 2021; Virani et al., 2021). As a gene-environment interacted disease, CHD is characterized by endothelial dysfunction and chronic inflammation, and is mainly caused by atherosclerosis which progresses slowly and is usually asymptomatic in the early stage (Hansson, 2005; Talmud, 2007; Gatto and Prati, 2020). Currently available biomarkers, such as high-sensitivity C-reactive protein (hsCRP), interleukin-6, myeloperoxidase (MPO), pregnancy-associated plasma protein-A (PAPP-A), myeloperoxidase, leukocyte counts, are inadequate for the diagnosis of CHD due to their poor clinical practice (Danesh et al., 2004; Lobbes et al., 2010; Wang et al., 2017; Li et al., 2018). Recent studies have proposed plasma metabolomics and micro-RNAs as potential biomarkers for the diagnosis of CHD, but further validations with a larger sample size are still needed (Wang et al., 2017; Zhang et al., 2018; Fu et al., 2019). Nevertheless, these biomarkers are inadequate for the detection of early CHD due to their insufficient clinical practice. The identification and development of novel biomarkers are necessary and urgent for the early detection of CHD.

The term epigenetics is defined as changes in gene expression without altering the DNA sequence itself. Epigenetic silencing can mimic genetic mutations by impairing the expression of a gene, and aberrant epigenetic signatures are known as disease-related (Laird, 2003). DNA methylation is one of the most important epigenetic signatures, having critical roles in the control of gene activities and the architecture of the nucleus of the cells (Robertson and Wolffe, 2000; Weber et al., 2005). Unlike mutations and other genetic abnormalities, epigenetic modifications are reversible and could be modified by lifestyles and therapeutic methods (Arasaradnam et al., 2008). In recent years, epigenetic aspects are believed to play a significant role in cardiovascular biology with various epigenetic mechanisms involved in the physiological and pathophysiological vascular differentiation, proliferation, and related inflammatory processes (Schleithoff et al., 2012). In addition, the associations between CHD and blood-based hypermethylation of several genes, such as *FOXP3* (forkhead box P3), *ABCG1* (ATP binding cassette subfamily G member 1) and *GALNT2* (polypeptide N-acetylgalactosaminyltransferase 2), and hypomethylation of *IL-6* (interleukin 6) have been reported (Jia et al., 2013; Peng et al., 2014; Zuo et al., 2016). Therefore, DNA methylation in blood could be a potential biomarker for the detection of CHD.

Protease-activated receptors (PARs) are a group of receptors that could promote inflammation in intimal tissue, enhance the initiation of atherosclerotic plaques (Coughlin, 2000), and induce vascular smooth muscle proliferation, migration, and collagen synthesis leading to plaque progression (Bretschneider et al., 1999; Wei et al., 2019). Also, PAR-mediated platelet activation may play a significant role in the plaque complications (thrombosis) and allows adhesion to atherosclerotic lesions, involving in the recruitment of monocytes and lymphocytes, and thus undermines plaque stability (Vorchheimer and Becker, 2006). Consequently, arteries may be blocked, leading to acute ischemic events such as acute coronary syndrome (ACSS), stroke, and transient ischemic attack (Vorchheimer and Becker, 2006). There are four known PARs subtypes PAR-1, PAR-2, PAR-3, and PAR-4, which are expressed in various cell types of the cardiovascular system including platelets, endothelial cells, and smooth muscle cells (Coughlin, 2000; Leger et al., 2006). PAR4, coded by the *F2RL3* gene (coagulation factor II receptor-like 3), is a member of the protease-activated receptor subfamily and is known to be expressed in the leukocytes (Vergnolle et al., 2002). Evidence shows that over-expression of the wild-type PAR4 is correlated with a higher sensitivity of cardiomyocytes to apoptosis (Kolpakov et al., 2016). Breitling et al. (2011) first disclosed decreased methylation of cg03636183 at *F2RL3* in the blood of heavy smokers. Later, one prospective study indicated the hypomethylation of blood-based *F2RL3* at cg03636183 cytidine-phosphate-guanosine (CpG) loci and five adjacent CpG sites upstream to be strongly related to the mortality among patients with stable coronary heart disease (Breitling et al., 2012). Another prospective cohort study has revealed the association between increased mortality of cardiovascular disease (CVD, defined by either physician-reported coronary heart disease or a self-reported history of myocardial infarction, stroke, pulmonary embolism, or revascularization of coronary arteries) and the hypomethylation of blood-based *F2RL3* at the cg03636183 CpG loci and other three flanking CpG sites upstream (Zhang et al., 2014b). An epigenome-wide study further suggested that the methylation levels of *F2RL3* not only at the cg03636183 loci but also at the cg24704287 loci were associated with cardiovascular disease mortality (Zhang et al., 2017). Follow-up studies, however, have not been reported about the diagnostic value of *F2RL3* methylation for CHD in any population, especially for early CHD.

To investigate the relationship between CHD and the blood-derived methylation of *F2RL3* in the Chinese population, we hereby performed a case-control study with 180 CHD patients and 184 healthy individuals, aiming to evaluate the associations between the methylation intensities and the status of CHD diseases, lifestyles, and historical treatments. Two amplicons based on cg03636183 and cg24704287 respectively were designed by EpiDesigner and analyzed by mass spectrometry.

## Materials and Methods

### Study Population

A total of 180 patients with CHD and 184 controls were collected from the Chinese PLA General Hospital from 2018 to 2019. All the CHD cases were confirmed according to the coronary angiography of the disease combined with clinical manifestations. Among the 180 CHD cases, 78 had MI, and 145 experienced heart failure. New York Heart Association (NYHA) (Yancy et al., 2017) cardiac function classifications were available from 161 CHD cases (NYHA Ⅰ CHD cases = 46; NYHA Ⅱ CHD cases = 78; NYHA Ⅲ CHD cases = 31; NYHA Ⅳ CHD cases = 6). CHD-free participants who participated in an annual health examination were randomly selected as controls. CHD cases and controls were matched by gender. The median age of patients with CHD was 66 years (58–73 years). Since the controls were recruited from the health examination center where most participants were under 70 years old, and thus the median age of controls was 63 years (57–68 years). All controls were self-report healthy, without a history of CHD, cancer, autoimmune diseases, and had normal blood accounts. No further exclusion or inclusion criteria were implemented for the controls. The detailed clinical characteristics of CHD cases and controls are listed in Table 1.

**TABLE 1:** Baseline characteristics of coronary heart disease (CHD) patients and healthy controls.

Clinical characteristics	Group	Controls (*N* = 184)	CHD cases (*N* = 180)	χ^2^	*p-*value^a^
		*N* (%)	N (%)		
Gender	Female	70 (38.0)	71 (39.4)		
	Male	114 (62.0)	109 (60.6)	0.08	0.784
Smoking	No	127 (69.0)	107 (59.4)		
	Yes	53 (28.8)	73 (40.6)		
	Unknown	4 (2.2)	0 (0.0)	4.88	**0.027**
Drinking	No	114 (61.9)	128 (71.1)		
	Yes	66 (35.9)	52 (28.9)		
	Unknown	4 (2.2)	0 (0.0)	2.47	0.116
Hypertension	No	93 (50.5)	50 (27.8)		
	Yes	84 (45.7)	130 (72.2)		
	Unknown	7 (3.8)	0 (0.0)	22.79	**2.00E-06**
Diabetes	No	132 (71.7)	118 (65.6)		
	Yes	45 (24.5)	62 (34.4)		
	Unknown	7 (3.8)	0 (0.0)	3.46	0.063

Clinical characteristics	Controls (*N* = 184)	CHD cases (*N* = 180)	Z	*p-*value^b^
	Median (IQR)	Median (IQR)		
Age	63 (57–68)	66 (58–73)	−2.67	**0.008**
Total cholesterol (TC) (mmol/L)	4.26 (3.61–5.06)	3.81 (3.25–4.42)	−3.41	**0.001**
Triglyceride (TG) (mmol/L)	1.39 (1.06–2.17)	1.30 (0.95–1.86)	−1.45	0.147
High density lipoprotein (HDL) (mmol/L)	1.15 (0.91–1.36)	1.09 (0.91–1.31)	−1.06	0.290
Low density lipoprotein (LDL) (mmol/L)	2.69 (2.06–3.40)	2.28 (1.82–2.87)	−3.70	**2.00E-04**

a The p-values were calculated by the Chi-square test, and significant p-values are in bold.

b The p-values were calculated by the Mann-Whitney test, and significant p-values are in bold.

### Sample Collection and Processing

Peripheral whole blood from CHD cases and healthy controls were deposited into the ethylene diamine tetraacetic acid (EDTA) tubes and kept at 4°C for up to 8 h before storing at −80°C till further usage. Genomic DNA was extracted from each sample using the Genomic DNA Extraction Kit (Zymo Research, Orange County, United States). Subsequently, DNA was bisulfite converted by the EZ-96 DNA Methylation Gold Kit according to the manufacturer’s instruction (Zymo Research, Orange County, United States).

### Agena Matrix-Assisted Laser Desorption Ionization Time-of-Flight (MALDI-TOF) Mass Spectrometry

Agena MALDI-TOF mass spectrometry (Agena Bioscience, San Diego, California, United States) described by Yang et al. (2015), was used for the quantification of DNA methylation levels. Procedures of methylation assessment and quality controls have been described previously (Zhang et al., 2014b). The cg03636183 and cg24704287 loci reported by Zhang et al. (2017), are located at 19p13.11 (chr19:17,000,586, at the second exon of *F2RL3*) and 19p13.13 (chr19:13,951,482, at the 5′ upstream of *F2RL3*), respectively. We therefore designed two amplicons: F2RL3_A amplicon (206 bp, chr19:17,000,421-17,000,626) covers CpG cg03636183; F2RL3_B amplicon (377 bp, chr19:13951024-13951400) covers five adjacent CpG sites of cg24704287 since the amplicons covering cg24704287 are unstable for PCR. The schematic diagram and the sequence of amplicons are presented in Supplementary Figure S1. SNPs are located neither at the primer regions nor overlapped with any CpGs in the two amplicons (F2RL3_A, F2RL3_B). The EpiTyper assay determined the methylation levels of 7 CpGs in F2RL3_A amplicon and yielded 7 distinguishable mass peaks, and determined the methylation levels of 5 CpGs in F2RL3_B amplicon and yielded 4 distinguishable mass peaks. F2RL3_B_CpG_4 and F2RL3_B_CpG_5 are located at the same fragment after the EpiTyper treatment, and thus the mass peak shows the average methylation level of F2RL3_B_CpG_4 and F2RL3_B_CpG_5 (presented as F2RL3_B_CpG_4.5). Briefly, the bisulfite-converted DNA was amplified by bisulfite-specific primers. The polymerase chain reaction (PCR) products were treated in the light of the standard protocol of Agena EpiTyper Assay by shrimp alkaline phosphatase (SAP) treatment and RNAse A cleavage (so-called “T-cleavage”) reaction. The samples were further cleaned by resin and then dispensed to a 384 SpectroCHIP using Nanodispenser. The chips were read by a MassARRAY system. Data were obtained by Spectro ACQUIRE v3.3.1.3 software and visualized with MassARRAY EpiTyper v1.2 software. For each batch of MassARRAY analysis, an equal number of cases and controls were treated and analyzed in parallel in all the processes.

### Statistical Analyses

All the statistical analyses were conducted by SPSS Statistics 25. Spearman’s rank correlation coefficient was carried out to evaluate the correlations. Differences between cases and controls were tested by non-parametric tests. ORs and 95% confidence intervals (CIs) were estimated by logistic regression models adjusted for covariates, especially for the significant covariates as indicated in Table 1. Cardiovascular-related quantitative variables are classified using appropriate cutoff values (Jellinger et al., 2017), including TC (5.0 mmol/L), TG (1.7 mmol/L), HDL (1.0 mmol/L), and LDL (3.0 mmol/L). ROC curve analysis was performed to assess the discriminatory power of altered *F2RL3* methylation levels for the diagnosis of CHD. The corresponding area under curve was calculated with 95% CIs. The statistical power was calculated by independent *t*-test using Power and Simple size software (http://powerandsamplesize.com/). All statistical tests were two-sided, and *p*-values less than 0.05 were defined as statistically significant.

## Results

### Blood-Based *F2RL3* Hypomethylation is Associated With CHD

**FIGURE 1:**
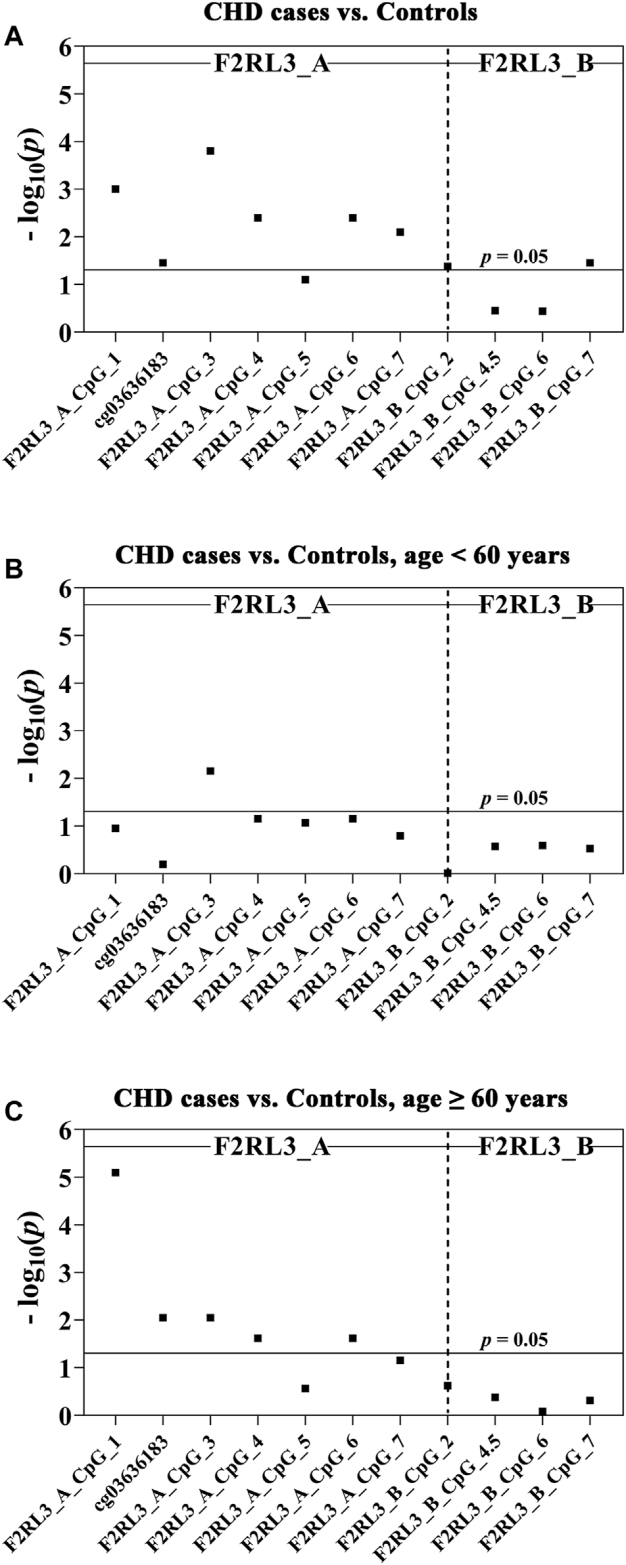
The association between CHD and decreased *F2RL3* methylation in the peripheral blood. The point plots show the methylation differences of CpG sites in the amplicon of F2RL3_A and F2RL3_B **(A)** between 184 controls and 180 CHD cases, **(B)** between 51 controls and 53 CHD cases, age <60 years, **(C)** between 133 controls and 127 CHD cases, age ≥60 years. The *p*-values of all the 12 measurable CpG loci in the two amplicons were calculated by logistic regression adjusted for age, gender, smoking, hypertension, TC, LDL, and batch effect. The vertical dashed line separates the two amplicons. The solid lines indicate the thresholds of *p*-value = 0.05.

In this study, we quantitatively determined the methylation levels of *F2RL3* in the blood DNA of the 180 CHD patients and 184 controls using Agena MALDI-TOF (matrix-assisted laser desorption ionization time-of-flight) mass spectrometry. Two amplicons in *F2RL3*, namely F2RL3_A amplicon (harboring seven measurable CpG sites) and F2RL3_B amplicon (harboring five measurable CpG sites), were amplified and analyzed. Three logistic regression models adjusted for different covariants were performed to investigate the association between *F2RL3* methylation and the status of CHD (Table 2). Among which, all the baseline characteristics that had significant differences between the CHD cases and the controls (as listed in Table 1) were adjusted in the logistic regression model 3. Six out of the seven CpG loci in the F2RL3_A amplicon showed significantly lower methylation in the CHD cases than in the controls according to the logistic regression model 3 (Odds ratios (ORs) per -10% methylation ranging from 1.22 to 1.42, *p* < 0.035 for all by logistic regression adjusted for age, gender, smoking, hypertension, total cholesterol (TC) levels, low density lipoprotein (LDL) levels and batch effect; Figure 1A and Table 2A). Among the significant CpG loci, F2RL3_A_CpG_3 was the most significant one, and F2RL3_A_CpG_2/cg03636183 was the weakest (Figure 1A and Table 2A). Weak associations were also observed between two out of the five measurable CpG sites in the F2RL3_B amplicon and the CHD (F2RL3_B_CpG_2, ORs per -10% methylation = 0.86, *p*-value = 0.042; F2RL3_B_CpG_7, ORs per -10% methylation = 1.92, *p*-value = 0.035, logistic regression model 3; Figure 1A and Table 2A). The power for methylation difference is sufficient (power for F2RL3_A_CpG_1, F2RL3_A_CpG_3, F2RL3_A_CpG_4, F2RL3_A_CpG_5, F2RL3_A_CpG_6, F2RL3_A_CpG_7, F2RL3_B_CpG_2 and F2RL3_B_CpG_7 was 0.9003, 0.9954, 0.9721, 0.7826, 0.9721, 0.9838, 0.8739 and 0.9893, respectively). We also noticed that the methylation correlates better among close than among more distant CpG. More specific, the methylation correlates better among CpGs in the same amplicon than CpGs in different amplicons which have larger distance (Supplementary Figure S2). In addition, the methylation correlation among the CpG sites in the F2RL3_A amplicon is stronger than the correlation among the CpG sites in the F2RL3_B amplicon (Supplementary Figure S2).

**TABLE 2:** Overall and age-specific methylation difference of *F2RL3* comparing CHD cases and controls.

A. Overall								
CpG sites	Controls (N = 184)	CHD cases (N = 180)	Model 1^a^		Model 2^b^		Model 3^c^	
	Median (IQR)	Median (IQR)	OR (95%CI) per-10% methylation	*p-*value	OR (95%CI) per-10% methylation	*p-*value	OR (95%CI) per-10% methylation	*p-*value
F2RL3_A_CpG_1	0.71 (0.49–0.82)	0.62 (0.46–0.78)	1.12 (1.01–1.24)	**0.028**	1.12 (1.01–1.24)	**0.029**	1.22 (1.08–1.38)	**0.001**
F2RL3_A_CpG_2/cg03636183	0.83 (0.79–0.87)	0.82 (0.75–0.87)	1.22 (0.98–1.53)	0.078	1.23 (0.98–1.55)	0.079	1.32 (1.02–1.71)	**0.035**
F2RL3_A_CpG_3	0.71 (0.66–0.77)	0.71 (0.55–0.79)	1.29 (1.11–1.51)	**0.001**	1.33 (1.13–1.55)	**4.28E-04**	1.41 (1.18–1.68)	**1.57E-04**
F2RL3_A_CpG_4	0.66 (0.59–0.70)	0.62 (0.55–0.68)	1.34 (1.09–1.65)	**0.005**	1.36 (1.10–1.68)	**0.005**	1.42 (1.12–1.81)	**0.004**
F2RL3_A_CpG_5	0.86 (0.82–0.89)	0.85 (0.76–0.90)	1.25 (1.00–1.56)	0.053	1.24 (0.99–1.57)	0.064	1.26 (0.97–1.63)	0.080
F2RL3_A_CpG_6	0.66 (0.59–0.70)	0.62 (0.55–0.68)	1.34 (1.09–1.65)	**0.005**	1.36 (1.10–1.68)	**0.005**	1.42 (1.12–1.81)	**0.004**
F2RL3_A_CpG_7	0.69 (0.58–0.75)	0.66 (0.52–0.72)	1.26 (1.06–1.48)	**0.007**	1.27 (1.07–1.50)	**0.007**	1.29 (1.07–1.56)	**0.008**
F2RL3_B_CpG_2	0.67 (0.57–0.81)	0.71 (0.63–0.82)	0.89 (0.78–1.00)	**0.048**	0.87 (0.77–0.99)	**0.029**	0.86 (0.75–0.99)	**0.042**
F2RL3_B_CpG_4.5	0.10 (0.08–0.14)	0.10 (0.06–0.14)	1.27 (0.91–1.78)	0.163	1.23 (0.88–1.74)	0.229	1.16 (0.85–1.58)	0.354
F2RL3_B_CpG_6	0.45 (0.39–0.50)	0.44 (0.36–0.51)	1.11 (0.94–1.31)	0.206	1.10 (0.93–1.30)	0.272	1.09 (0.91–1.30)	0.365
F2RL3_B_CpG_7	0.04 (0.02–0.07)	0.04 (0.01–0.06)	1.94 (1.11–3.38)	**0.020**	1.97 (1.12–3.47)	**0.019**	1.92 (1.05–3.54)	**0.035**

B. Age < 60 years								
CpG sites	Controls (*N* = 51)	CHD cases (*N* = 53)	Model 1 ^a^		Model 2^b^		Model 3^c^	
	Median (IQR)	Median (IQR)	OR (95%CI) per-10% methylation	*p-*value	OR (95%CI) per-10% methylation	*p-*value	OR (95%CI) per-10% methylation	*p-*value
F2RL3_A_CpG_1	0.54 (0.43–0.70)	0.72 (0.47–0.82)	0.84 (0.70–1.02)	0.081	0.85 (0.70–1.04)	0.105	0.82 (0.64–1.05)	0.111
F2RL3_A_CpG_2/cg03636183	0.84 (0.78–0.88)	0.84 (0.76–0.88)	1.13 (0.76–1.67)	0.551	1.08 (0.72–1.61)	0.711	1.13 (0.69–1.84)	0.634
F2RL3_A_CpG_3	0.72 (0.67–0.78)	0.71 (0.45–0.79)	1.36 (1.05–1.76)	**0.019**	1.34 (1.03–1.74)	**0.029**	1.61 (1.14–2.29)	**0.007**
F2RL3_A_CpG_4	0.67 (0.60–0.72)	0.63 (0.57–0.69)	1.44 (0.98–2.10)	0.064	1.38 (0.92–2.07)	0.119	1.57 (0.96–2.56)	0.070
F2RL3_A_CpG_5	0.86 (0.82–0.90)	0.85 (0.78–0.90)	1.48 (0.94–2.35)	0.094	1.44 (0.90–2.30)	0.124	1.66 (0.93–2.95)	0.085
F2RL3_A_CpG_6	0.67 (0.60–0.72)	0.63 (0.57–0.69)	1.44 (0.98–2.10)	0.064	1.38 (0.92–2.07)	0.119	1.57 (0.96–2.56)	0.070
F2RL3_A_CpG_7	0.68 (0.59–0.76)	0.67 (0.52–0.72)	1.30 (0.96–1.75)	0.090	1.25 (0.91–1.73)	0.173	1.34 (0.89–2.00)	0.159
F2RL3_B_CpG_2	0.77 (0.69–0.85)	0.70 (0.63–0.82)	1.01 (0.82–1.24)	0.947	1.02 (0.82–1.26)	0.882	1.01 (0.65–1.57)	0.964
F2RL3_B_CpG_4.5	0.12 (0.07–0.15)	0.10 (0.08–0.14)	1.20 (0.77–1.87)	0.433	1.18 (0.77–1.82)	0.451	1.34 (0.80–2.27)	0.267
F2RL3_B_CpG_6	0.47 (0.40–0.57)	0.44 (0.37–0.49)	1.24 (0.95–1.62)	0.120	1.23 (0.93–1.62)	0.144	1.36 (0.80–2.32)	0.255
F2RL3_B_CpG_7	0.05 (0.02–0.07)	0.03 (0.00–0.06)	3.16 (1.10–9.13)	**0.033**	2.88 (0.97–8.56)	0.058	1.54 (0.68–3.48)	0.296

C. Age ≥ 60 years								
CpG sites	Controls (*N* = 133)	CHD cases (*N* = 127)	Model 1^a^		Model 2^b^		Model 3^c^	
	Median (IQR)	Median (IQR)	OR (95%CI) per-10% methylation	*p-*value	OR (95%CI) per-10% methylation	*p-*value	OR (95%CI) per-10% methylation	*p-*value
F2RL3_A_CpG_1	0.76 (0.57–0.83)	0.61 (0.45–0.78)	1.27 (1.12–1.44)	**2.58E-04**	1.26 (1.10–1.44)	**0.001**	1.49 (1.25–1.77)	**8.00E-06**
F2RL3_A_CpG_2/cg03636183	0.83 (0.79–0.87)	0.81 (0.74–0.86)	1.28 (0.97–1.68)	0.079	1.42 (1.05–1.90)	**0.021**	1.58 (1.12–2.21)	**0.009**
F2RL3_A_CpG_3	0.70 (0.66–0.77)	0.71 (0.58–0.80)	1.25 (1.03–1.52)	**0.025**	1.31 (1.07–1.61)	**0.009**	1.35 (1.08–1.70)	**0.009**
F2RL3_A_CpG_4	0.65 (0.59–0.70)	0.62 (0.54–0.68)	1.31 (1.03–1.67)	**0.030**	1.37 (1.05–1.78)	**0.021**	1.41 (1.05–1.91)	**0.024**
F2RL3_A_CpG_5	0.85 (0.81–0.89)	0.85 (0.76–0.89)	1.18 (0.92–1.52)	0.198	1.22 (0.93–1.61)	0.158	1.18 (0.88–1.59)	0.275
F2RL3_A_CpG_6	0.65 (0.59–0.70)	0.62 (0.54–0.68)	1.31 (1.03–1.67)	**0.030**	1.37 (1.05–1.78)	**0.021**	1.41 (1.05–1.91)	**0.024**
F2RL3_A_CpG_7	0.69 (0.58–0.75)	0.66 (0.52–0.72)	1.24 (1.02–1.51)	**0.034**	1.24 (1.00–1.54)	**0.046**	1.24 (0.98–1.57)	0.070
F2RL3_B_CpG_2	0.62 (0.55–0.77)	0.72 (0.62–0.82)	0.83 (0.72–0.97)	**0.017**	0.83 (0.71–0.97)	**0.018**	0.90 (0.75–1.07)	0.238
F2RL3_B_CpG_4.5	0.10 (0.08–0.13)	0.09 (0.06–0.12)	1.37 (0.86–2.17)	0.184	1.34 (0.83–2.18)	0.237	1.24 (0.74–2.10)	0.417
F2RL3_B_CpG_6	0.44 (0.38–0.49)	0.44 (0.35–0.52)	1.04 (0.84–1.29)	0.722	0.98 (0.78–1.24)	0.881	1.03 (0.80–1.32)	0.825
F2RL3_B_CpG_7	0.04 (0.02–0.07)	0.04 (0.01–0.06)	1.55 (0.80–3.03)	0.198	1.39 (0.70–2.79)	0.350	1.31 (0.61–2.81)	0.489

a Model 1: Logistic regression without adjustment.

b Model 2: Logistic regression adjusted for age and gender.

c Model 3: Logistic regression adjusted for age, gender, smoking, hypertension, TC, LDL, and batch effect. Significant p-values are in bold.

The level of methylation has been known to be changed along with age (Horvath and Raj, 2018). We hereby stratified the subjects by the age of 60 years old. In the group younger than 60 years old, only one CpG site (F2RL3_A_CpG_3) was weakly associated with the CHD, whereas the other 11 measurable CpG sites showed no correlation (Figure 1B and Table 2B). In the group ≥60 years old, five out of the seven measurable CpG loci in F2RL3_A amplicon exhibited significantly lower methylation levels in the CHD cases than in the controls. Among which, F2RL3_A_CpG_1 showed the most significant difference (OR per -10% methylation = 1.49, *p* = 8.00 × 10^−6^ by logistic regression model 3; Figure 1C and Table 2C). In the F2RL3_B amplicon, none of the five measurable CpG loci indicated any association with CHD in people older than 60 years old (Figure 1C and Table 2C). When the subjects were stratified by 65 years old, we also found a similar pattern of the age-dependent *F2RL3* methylation for the risk of CHD (Supplementary Table S1). Moreover, the hypomethylation of F2RL3_A CpG sites showed even larger ORs for per -10% methylation in the group ≥65 years old than in the group ≥60 years old (Table 2C and Supplementary Table 1B), suggesting that age is a cofounder of the *F2RL3* hypomethylation associated risk for the CHD.

### Decreased *F2RL3* Methylation is Mainly Associated With MI

**FIGURE 2:**
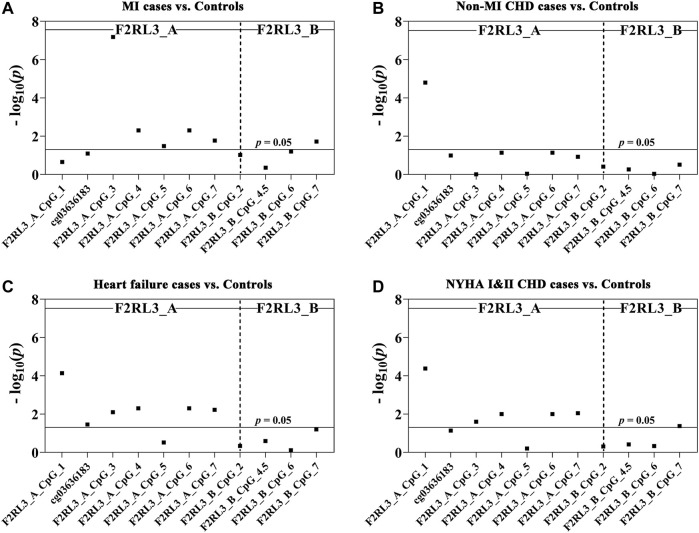
The association between decreased *F2RL3* methylation in the peripheral blood and MI cases, non-MI CHD cases, heart failure cases, and patients with minor to medium cardiac function impairment (NYHA Ⅰ&Ⅱ CHD cases). The point plots show the methylation differences of CpG sites in the amplicon of F2RL3_A and F2RL3_B **(A)** between 184 controls and 78 MI cases, **(B)** between 184 controls and 102 non-MI CHD cases, **(C)** between 184 controls and 145 heart failure cases, **(D)** between 184 controls and 124 NYHA Ⅰ&Ⅱ CHD cases. The *p*-values of all the 12 measurable CpG loci in the two amplicons were calculated by logistic regression adjusted for age, gender, smoking, hypertension, TC, LDL, and batch effect. The vertical dashed line separates the two amplicons. The solid lines indicate the thresholds of *p*-value = 0.05.

Among the 180 CHD patients, 78 experienced myocardial infarction (MI). Thus, we further investigated whether MI played a role in the CHD associated *F2RL3* methylation in the blood. The F2RL3_A_CpG_3 site showed the most significant hypomethylation in the MI cases than in the controls (MI cases: median (interquartile range (IQR)) = 0.56 (0.44–0.80); controls: median (IQR) = 0.71 (0.66–0.77); OR per -10% methylation = 2.00, *p* = 6.59 × 10^−8^ by logistic regression adjusted for age, gender, smoking, hypertension, TC levels, LDL levels and batch effect; Figure 2A and Table 3A). Four additional CpG sites in the F2RL3_A amplicon located at the upstream of F2RL3_A_CpG_3 also exhibited significantly lower methylation levels in the MI cases than in the controls (OR per -10% methylation ranging from 1.32 to 1.51, *p* < 0.033 for all by logistic regression adjusted for covariant; Figure 2A and Table 3A). The methylation levels of F2RL3_A_CpG_1 and F2RL3_A_CpG_2/cg03636183 were also lower in the MI cases than in the controls but without significance (Figure 2A and Table 3A). In the F2RL3_B amplicon, only one out of the five measurable CpG sites showed significant association with MI (F2RL3_B_CpG_7, ORs per -10% methylation = 2.99, *p* = 0.019 by logistic regression adjusted for covariant; Figure 2A and Table 3A). We further evaluated the *F2RL3* methylation difference between the non-MI CHD cases and the controls. Interestingly, unlike the MI cases, the only altered methylation for the non-MI CHD cases compared to the controls was detected in the F2RL3_A_CpG_1 site (OR per -10% methylation = 1.44, *p* = 1.60 × 10^−5^ by logistic regression adjusted for covariant; Figure 2B and Table 3B).

**TABLE 3:** *F2RL3* methylation in MI cases, non-MI CHD cases, heart failure cases, and patients with minor to medium cardiac function impairment (NYHA Ⅰ&Ⅱ CHD cases) compared to controls.

A. MI cases vs. controls								
CpG sites	Controls (*N* = 184)	MI cases (*N* = 78)	Model 1^a^		Model 2^b^		Model 3^c^	
	Median (IQR)	Median (IQR)	OR (95%CI) per-10% methylation	*p-*value	OR (95%CI) per-10% methylation	*p-*value	OR (95%CI) per-10% methylation	*p-*value
F2RL3_A_CpG_1	0.71 (0.49–0.82)	0.71 (0.46–0.80)	1.06 (0.94–1.21)	0.340	1.07 (0.94–1.22)	0.303	1.10 (0.94–1.28)	0.223
F2RL3_A_CpG_2/cg03636183	0.83 (0.79–0.87)	0.82 (0.73–0.86)	1.39 (1.06–1.82)	**0.017**	1.28 (0.97–1.69)	0.079	1.31 (0.97–1.78)	0.081
F2RL3_A_CpG_3	0.71 (0.66–0.77)	0.56 (0.44–0.80)	1.72 (1.39–2.12)	**4.73E-07**	1.71 (1.39–2.12)	**6.77E-07**	2.00 (1.55–2.57)	**6.59E-08**
F2RL3_A_CpG_4	0.66 (0.59–0.70)	0.61 (0.48–0.68)	1.57 (1.22–2.02)	**0.001**	1.44 (1.11–1.87)	**0.006**	1.51 (1.13–2.02)	**0.005**
F2RL3_A_CpG_5	0.86 (0.82–0.89)	0.85 (0.71–0.89)	1.49 (1.14–1.96)	**0.004**	1.39 (1.06–1.82)	**0.019**	1.39 (1.03–1.88)	**0.033**
F2RL3_A_CpG_6	0.66 (0.59–0.70)	0.61 (0.48–0.68)	1.57 (1.22–2.02)	**0.001**	1.44 (1.11–1.87)	**0.006**	1.51 (1.13–2.02)	**0.005**
F2RL3_A_CpG_7	0.69 (0.58–0.75)	0.61 (0.46–0.71)	1.41 (1.15–1.74)	**0.001**	1.33 (1.07–1.64)	**0.009**	1.32 (1.05–1.67)	**0.017**
F2RL3_B_CpG_2	0.67 (0.57–0.81)	0.69 (0.61–0.78)	0.93 (0.79–1.08)	0.335	0.92 (0.78–1.08)	0.310	0.85 (0.71–1.03)	0.096
F2RL3_B_CpG_4.5	0.10 (0.08–0.14)	0.08 (0.06–0.14)	1.38 (0.85–2.22)	0.193	1.26 (0.80–2.00)	0.319	1.18 (0.78–1.77)	0.441
F2RL3_B_CpG_6	0.45 (0.39–0.50)	0.40 (0.29–0.49)	1.29 (1.03–1.61)	**0.025**	1.27 (1.02–1.59)	**0.035**	1.24 (0.99–1.56)	0.063
F2RL3_B_CpG_7	0.04 (0.02–0.07)	0.03 (0.00–0.06)	2.98 (1.32–6.69)	**0.008**	2.68 (1.15–6.23)	**0.022**	2.99 (1.20–7.47)	**0.019**

B. non-MI CHD cases vs. controls								
CpG sites	Controls (*N* = 184)	non-MI CHD cases (*N* = 102)	Model 1^a^		Model 2**^b^**		Model 3^c^	
	Median (IQR)	Median (IQR)	OR (95%CI) per-10% methylation	*p-*value	OR (95%CI) per-10% methylation	*p-*value	OR (95%CI) per-10% methylation	*p-*value
F2RL3_A_CpG_1	0.71 (0.49–0.82)	0.59 (0.47–0.76)	1.18 (1.04–1.33)	**0.011**	1.22 (1.07–1.39)	**0.003**	1.44 (1.22–1.70)	**1.60E-05**
F2RL3_A_CpG_2/cg03636183	0.83 (0.79–0.87)	0.83 (0.77–0.87)	1.10 (0.83–1.47)	0.504	1.17 (0.86–1.58)	0.314	1.35 (0.94–1.92)	0.102
F2RL3_A_CpG_3	0.71 (0.66–0.77)	0.73 (0.63–0.79)	1.04 (0.84–1.31)	0.704	1.03 (0.82–1.30)	0.776	1.00 (0.77–1.29)	0.980
F2RL3_A_CpG_4	0.66 (0.59–0.70)	0.65 (0.57–0.69)	1.19 (0.92–1.53)	0.183	1.21 (0.92–1.59)	0.165	1.33 (0.97–1.82)	0.073
F2RL3_A_CpG_5	0.86 (0.82–0.89)	0.85 (0.80–0.90)	1.04 (0.79–1.36)	0.798	1.02 (0.76–1.35)	0.914	1.02 (0.75–1.38)	0.920
F2RL3_A_CpG_6	0.66 (0.59–0.70)	0.65 (0.57–0.69)	1.19 (0.92–1.53)	0.183	1.21 (0.92–1.59)	0.165	1.33 (0.97–1.82)	0.073
F2RL3_A_CpG_7	0.69 (0.58–0.75)	0.67 (0.55–0.73)	1.15 (0.94–1.41)	0.169	1.18 (0.94–1.47)	0.151	1.22 (0.95–1.56)	0.119
F2RL3_B_CpG_2	0.67 (0.57–0.81)	0.73 (0.65–0.83)	0.86 (0.74–0.99)	**0.035**	0.83 (0.71–0.96)	**0.014**	0.92 (0.77–1.11)	0.389
F2RL3_B_CpG_4.5	0.10 (0.08–0.14)	0.10 (0.07–0.13)	1.21 (0.81–1.81)	0.346	1.21 (0.77–1.89)	0.410	1.12 (0.77–1.64)	0.548
F2RL3_B_CpG_6	0.45 (0.39–0.50)	0.47 (0.38–0.52)	0.99 (0.80–1.24)	0.953	0.96 (0.76–1.21)	0.714	1.01 (0.78–1.31)	0.933
F2RL3_B_CpG_7	0.04 (0.02–0.07)	0.04 (0.02–0.06)	1.51 (0.81–2.81)	0.197	1.50 (0.78–2.88)	0.225	1.37 (0.75–2.51)	0.308

C. Heart failure cases vs. controls								
CpG sites	Controls (*N* = 184)	Heart failure cases (*N* = 145)	Model 1^a^		Model 2^b^		Model 3^c^	
	Median (IQR)	Median (IQR)	OR (95%CI) per-10% methylation	*p-*value	OR (95%CI) per-10% methylation	*p-*value	OR (95%CI) per-10% methylation	*p-*value
F2RL3_A_CpG_1	0.71 (0.49–0.82)	0.56 (0.42–0.78)	1.20 (1.07–1.33)	**0.001**	1.20 (1.08–1.34)	**0.001**	1.30 (1.14–1.47)	**7.30E-05**
F2RL3_A_CpG_2/cg03636183	0.83 (0.79–0.87)	0.82 (0.76–0.86)	1.24 (0.98–1.58)	0.079	1.23 (0.96–1.58)	0.103	1.35 (1.02–1.79)	**0.035**
F2RL3_A_CpG_3	0.71 (0.66–0.77)	0.72 (0.59–0.81)	1.17 (0.99–1.37)	0.064	1.21 (1.02–1.43)	**0.028**	1.29 (1.07–1.55)	**0.008**
F2RL3_A_CpG_4	0.66 (0.59–0.70)	0.62 (0.55–0.68)	1.35 (1.09–1.68)	**0.006**	1.34 (1.07–1.67)	**0.010**	1.43 (1.12–1.83)	**0.005**
F2RL3_A_CpG_5	0.86 (0.82–0.89)	0.85 (0.78–0.90)	1.17 (0.92–1.49)	0.198	1.14 (0.89–1.46)	0.313	1.16 (0.88–1.53)	0.304
F2RL3_A_CpG_6	0.66 (0.59–0.70)	0.62 (0.55–0.68)	1.35 (1.09–1.68)	**0.006**	1.34 (1.07–1.67)	**0.010**	1.43 (1.12–1.83)	**0.005**
F2RL3_A_CpG_7	0.69 (0.58–0.75)	0.66 (0.51–0.72)	1.31 (1.10–1.56)	**0.003**	1.30 (1.08–1.56)	**0.006**	1.33 (1.08–1.62)	**0.006**
F2RL3_B_CpG_2	0.67 (0.57–0.81)	0.70 (0.62–0.81)	0.91 (0.80–1.04)	0.149	0.90 (0.79–1.02)	0.106	0.94 (0.81–1.10)	0.462
F2RL3_B_CpG_4.5	0.10 (0.08–0.14)	0.10 (0.06–0.13)	1.34 (0.91–1.98)	0.137	1.26 (0.86–1.86)	0.239	1.22 (0.86–1.73)	0.257
F2RL3_B_CpG_6	0.45 (0.39–0.50)	0.45 (0.37–0.52)	1.05 (0.88–1.25)	0.580	1.02 (0.85–1.22)	0.840	1.03 (0.85–1.25)	0.779
F2RL3_B_CpG_7	0.04 (0.02–0.07)	0.04 (0.01–0.06)	1.99 (1.09–3.63)	**0.025**	1.95 (1.06–3.61)	**0.033**	1.85 (0.97–3.55)	0.063

D. NYHA Ⅰ&Ⅱ CHD cases vs. controls								
CpG sites	Controls (*N* = 184)	NYHA Ⅰ&Ⅱ CHD cases (*N* = 124)	Model 1^a^		Model 2^b^		Model 3^c^	
	Median (IQR)	Median (IQR)	OR (95%CI) per-10% methylation	*p-*value	OR (95%CI) per-10% methylation	*p-*value	OR (95%CI) per-10% methylation	*p-*value
F2RL3_A_CpG_1	0.71 (0.49–0.82)	0.56 (0.40–0.78)	1.21 (1.08–1.36)	**0.001**	1.23 (1.09–1.38)	**0.001**	1.35 (1.17–1.55)	**4.20E-05**
F2RL3_A_CpG_2/cg03636183	0.83 (0.79–0.87)	0.82 (0.77–0.87)	1.14 (0.88–1.47)	0.335	1.18 (0.90–1.55)	0.229	1.33 (0.97–1.81)	0.073
F2RL3_A_CpG_3	0.71 (0.66–0.77)	0.73 (0.61–0.82)	1.12 (0.95–1.33)	0.178	1.16 (0.97–1.38)	0.098	1.25 (1.03–1.53)	**0.025**
F2RL3_A_CpG_4	0.66 (0.59–0.70)	0.63 (0.57–0.68)	1.27 (1.01–1.60)	**0.045**	1.32 (1.03–1.68)	**0.027**	1.44 (1.09–1.89)	**0.010**
F2RL3_A_CpG_5	0.86 (0.82–0.89)	0.87 (0.80–0.90)	0.93 (0.71–1.22)	0.611	0.94 (0.71–1.24)	0.640	0.93 (0.69–1.25)	0.629
F2RL3_A_CpG_6	0.66 (0.59–0.70)	0.63 (0.57–0.68)	1.27 (1.01–1.60)	**0.045**	1.32 (1.03–1.68)	**0.027**	1.44 (1.09–1.89)	**0.010**
F2RL3_A_CpG_7	0.69 (0.58–0.75)	0.66 (0.53–0.72)	1.25 (1.04–1.50)	**0.018**	1.29 (1.07–1.57)	**0.009**	1.34 (1.08–1.66)	**0.009**
F2RL3_B_CpG_2	0.67 (0.57–0.81)	0.71 (0.62–0.81)	0.90 (0.78–1.03)	0.114	0.88 (0.76–1.01)	0.074	0.94 (0.79–1.12)	0.493
F2RL3_B_CpG_4.5	0.10 (0.08–0.14)	0.10 (0.07–0.14)	1.17 (0.82–1.67)	0.398	1.15 (0.79–1.66)	0.471	1.16 (0.83–1.64)	0.387
F2RL3_B_CpG_6	0.45 (0.39–0.50)	0.45 (0.39–0.51)	1.03 (0.85–1.25)	0.741	1.03 (0.84–1.25)	0.799	1.08 (0.88–1.34)	0.467
F2RL3_B_CpG_7	0.04 (0.02–0.07)	0.04 (0.01–0.06)	2.10 (1.11–4.00)	**0.024**	2.09 (1.09–4.00)	**0.026**	2.11 (1.03–4.31)	**0.042**

a Model 1: Logistic regression without adjustment.

b Model 2: Logistic regression adjusted for age and gender.

c Model 3: Logistic regression adjusted for age, gender, smoking, hypertension, TC, LDL, and batch effect. Significant p-values are in bold. MI, myocardial infarction; NYHA, new york heart association.

### The Difference of *F2RL3* Methylation Level Between Heart Failure Cases Versus Controls

There were 145 heart failure cases in the 180 CHD patients. Hence, we also investigated the association between *F2RL3* methylation and the status of heart failure (Table 3C). Here, six out of the seven CpG loci in the F2RL3_A amplicon revealed significantly lower methylation levels in the heart failure cases than in the controls (OR per -10% methylation ranging from 1.29 to 1.43, *p* < 0.035 for all by logistic regression adjusted for age, gender, smoking, hypertension, TC levels, LDL levels and batch effect; Figure 2C and Table 3C). Among the significant CpG loci, F2RL3_A_CpG_1 showed the most significant difference (OR per -10% methylation = 1.30, *p* = 7.30 × 10^−5^ by logistic regression adjusted for covariant; Figure 2C and Table 3C). In the F2RL3_B amplicon, on the contrary, none of the five measurable CpG loci displayed any association with heart failure (Figure 2C and Table 3C). Since there are only 35 CHD patients without heart failure, we did not investigate the *F2RL3* methylation difference between the non-heart failure CHD cases and the controls using logistic regression analysis.

### *F2RL3* Methylation Difference Between NYHA Ⅰ&Ⅱ CHD Cases Versus Controls

Among the 180 CHD patients, patients with minor to medium cardiac function impairment (NYHA Ⅰ&Ⅱ CHD cases) were available from 124 CHD cases (NYHA Ⅰ CHD cases = 46, NYHA Ⅱ CHD cases = 78). Compared to the healthy controls, the status of *F2RL3* methylation was also associated with NYHA Ⅰ&Ⅱ CHD cases. The methylation level of five out of the seven CpG loci in the F2RL3_A amplicon was also significantly decreased for NYHA Ⅰ&Ⅱ CHD cases with ORs >1.25 per -10% methylation (*p* < 0.025 for all by logistic regression adjusted for age, gender, smoking, hypertension, TC levels, LDL levels and batch effect; Figure 2D and Table 3D). Here, the most significant locus was F2RL3_A_CpG_1 with an OR of 1.35 per -10% methylation and a *p*-value of 4.20 × 10^−5^ (Figure 2D and Table 3D). Only one out of five measurable CpG sites in the F2RL3_B amplicon exhibited a weak significant association with the NYHA Ⅰ&Ⅱ CHD cases (F2RL3_B_CpG_7, ORs per -10% methylation = 2.11, *p* = 0.042, logistic regression adjusted for covariant; Figure 2D and Table 3D). Although the sample size of NYHA Ⅲ&Ⅳ CHD cases was very small (only 37 cases), to investigate the correlation between *F2RL3* methylation and the level of cardiac function impairment, logistic regression was also applied. Compared with the healthy controls, NYHA Ⅲ&Ⅳ CHD patients showed significantly decreased methylation in the F2RL3_A amplicon (Supplementary Table S2). To note, the ORs of all significant F2RL3_A loci in the NYHA Ⅲ&Ⅳ CHD patients were larger than that in the NYHA Ⅰ&II CHD patients (Figure 2D; Table 3D and Supplementary Table S2). Additionally, we attempted to explore the methylation differences between NYHA Ⅰ&II CHD cases and NYHA Ⅲ&Ⅳ CHD cases using the Mann-Whitney test. The NYHA Ⅲ&Ⅳ CHD cases showed significantly decreased methylation than the NYHA Ⅰ&II CHD cases at the sites of F2RL3_A_CpG_3, F2RL3_A_CpG_5, and F2RL3_B_CpG_4.5 (*p* < 0.032 for all; Supplementary Table S3). This indicated that the aberrant *F2RL3* methylation would be enhanced along with the impairment of cardiac function.

### *F2RL3* Methylation and CHD-Related Characteristics

To explore the relationship between the blood-based *F2RL3* methylation and the CHD-related characteristics, the subjects (including both CHD cases and controls) with available data were interpreted. In agreement with previous reports (Breitling et al., 2011; Wan et al., 2012; Sun et al., 2013; Zhang et al., 2014a), smoking has a tremendous influence on the *F2RL3* methylation especially at the F2RL3_A amplicon which covers F2RL3_A_CpG_2/cg036361837 and six flanking CpG sites (Table 4). In contrast, only one CpG locus in the F2RL3_B amplicon showed a borderline association with smoking in our study (Table 4). When stratified by the status of smoking, we unexpectedly found that the methylation of F2RL3_A amplicon was better associated with non-smokers than smokers with larger ORs and more significant *p*-values, whereas the methylation of F2RL3_B amplicon was associated with smokers with larger ORs than non-smokers **(** Supplementary Table S4 **)**. The males showed lower methylation levels than the females but also mainly in the F2RL3_A amplicon (Table 4). As shown in Table 4, weak methylation differences were observed in a few CpG sites when stratified by age groups, diabetes, TC levels, and high density lipoprotein (HDL) levels. The methylation levels of all the 12 CpG sites in the F2RL3_A amplicon and F2RL3_B amplicon showed no correlation with drinking, hypertension, levels of triglyceride (TG), and LDL (Table 4).

**TABLE 4:** The association between *F2RL3* methylation and CHD-related characteristics in the study subjects.

Characteristics (N)	Median (IQR) of methylation levels											
	Group (N)	F2RL3_A_CpG_1	F2RL3_A_CpG_2/cg036361837	F2RL3_A_CpG_3	F2RL3_A_CpG_4	F2RL3_A_CpG_5	F2RL3_A_CpG_6	F2RL3_A_CpG_7	F2RL3_B_CpG_2	F2RL3_B_CpG_4.5	F2RL3_B_CpG_6	F2RL3_B_CpG_7
Age (380)	<60 (110)	0.60 (0.44–0.78)	0.84 (0.76–0.88)	0.70 (0.58–0.78)	0.65 (0.58–0.70)	0.86 (0.80–0.90)	0.65 (0.58–0.70)	0.67 (0.55–0.74)	0.74 (0.64–0.82)	0.11 (0.07–0.15)	0.45 (0.39–0.52)	0.04 (0.01–0.07)
	≥60 (270)	0.71 (0.49–0.82)	0.83 (0.76–0.87)	0.70 (0.63–0.78)	0.64 (0.56–0.69)	0.85 (0.79–0.89)	0.64 (0.56–0.69)	0.67 (0.56–0.74)	0.68 (0.58–0.78)	0.10 (0.07–0.13)	0.44 (0.37–0.50)	0.04 (0.02–0.06)
	*p*-value*	**0.042**	0.209	0.600	0.436	0.366	0.436	0.936	**0.002**	0.124	0.427	0.325
Gender (380)	Female (152)	0.72 (0.49–0.82)	0.84 (0.80–0.87)	0.71(0.62–0.78)	0.67 (0.61–0.71)	0.87 (0.84–0.91)	0.67 (0.61–0.71)	0.68 (0.62–0.75)	0.69 (0.58–0.82)	0.10 (0.07–0.14)	0.45 (0.38–0.52)	0.04 (0.02–0.07)
	Male (228)	0.67 (0.48–0.81)	0.81 (0.74–0.86)	0.69 (0.63–0.77)	0.63 (0.53–0.68)	0.84 (0.77–0.89)	0.63 (0.53–0.68)	0.65 (0.52–0.72)	0.69 (0.60–0.80)	0.09 (0.07–0.13)	0.43 (0.37–0.50)	0.04 (0.01–0.06)
	*p*-value*	0.316	**1.22E-04**	0.329	**1.60E-05**	**6.00E-06**	**1.60E-05**	**7.20E-05**	0.651	**0.044**	0.178	0.669
Smoking (376)	No (248)	0.70 (0.49–0.82)	0.84 (0.79–0.87)	0.71 (0.63–0.77)	0.66 (0.61–0.70)	0.87 (0.83–0.90)	0.66 (0.61–0.70)	0.69 (0.62–0.75)	0.69 (0.59–0.80)	0.10 (0.07–0.14)	0.45 (0.38–0.50)	0.04 (0.02–0.07)
	Yes (128)	0.65 (0.44–0.80)	0.79 (0.72–0.85)	0.69 (0.62–0.79)	0.59 (0.51–0.65)	0.82 (0.75–0.87)	0.59 (0.51–0.65)	0.58 (0.48–0.69)	0.70 (0.61–0.81)	0.09 (0.06–0.13)	0.44 (0.36–0.51)	0.04 (0.01–0.06)
	*p*-value*	0.123	**8.72E-07**	0.943	**6.97E-11**	**2.76E-08**	**6.97E-11**	**1.46E-09**	0.508	**0.046**	0.677	0.422
Drinking (376)	No (254)	0.70 (0.49–0.82)	0.83 (0.77–0.87)	0.70 (0.61–0.77)	0.65 (0.59–0.69)	0.86 (0.80–0.90)	0.65 (0.59–0.69)	0.67 (0.57–0.74)	0.68 (0.58–0.79)	0.10 (0.07–0.14)	0.45 (0.37–0.51)	0.04 (0.02–0.07)
	Yes (122)	0.67 (0.47–0.81)	0.83 (0.75–0.86)	0.72 (0.65–0.79)	0.62 (0.57–0.70)	0.85 (0.79–0.89)	0.62 (0.57–0.70)	0.66 (0.53–0.73)	0.71 (0.62–0.83)	0.10 (0.06–0.13)	0.44 (0.38–0.50)	0.04 (0.01–0.07)
	*p*-value*	0.415	0.310	0.104	0.187	0.279	0.187	0.282	0.052	0.500	0.923	0.807
Hypertension (373)	No (148)	0.67 (0.48–0.79)	0.82 (0.74–0.87)	0.70 (0.63–0.78)	0.65 (0.57–0.70)	0.85 (0.79–0.89)	0.65 (0.57–0.70)	0.67 (0.56–0.74)	0.69 (0.60–0.80)	0.10 (0.07–0.14)	0.44 (0.38–0.52)	0.04 (0.01–0.07)
	Yes (225)	0.70 (0.49–0.82)	0.83 (0.78–0.87)	0.70 (0.62–0.78)	0.65 (0.57–0.69)	0.86 (0.80–0.90)	0.65 (0.57–0.69)	0.67 (0.56–0.73)	0.69 (0.59–0.81)	0.10 (0.07–0.13)	0.44 (0.36–0.50)	0.04 (0.02–0.06)
	*p*-value*	0.212	0.237	0.858	0.805	0.637	0.805	0.982	0.933	0.166	0.440	0.742
Diabetes (373)	No (264)	0.70 (0.49–0.81)	0.82 (0.76–0.86)	0.69 (0.62–0.77)	0.64 (0.57–0.70)	0.85 (0.79–0.89)	0.64 (0.57–0.70)	0.66 (0.55–0.74)	0.69 (0.61–0.81)	0.10 (0.07–0.13)	0.43 (0.37–0.50)	0.04 (0.01–0.07)
	Yes (109)	0.62 (0.45–0.82)	0.84 (0.79–0.88)	0.72 (0.65–0.79)	0.65 (0.59–0.69)	0.87 (0.81–0.90)	0.65 (0.59–0.69)	0.68 (0.59–0.74)	0.68 (0.57–0.79)	0.10 (0.07–0.14)	0.45 (0.36–0.51)	0.04 (0.02–0.06)
	*p*-value*	0.253	**0.017**	0.073	0.375	0.055	0.375	0.235	0.202	0.622	0.583	0.200
TC (376)	<5.0 mmol/L (296)	0.70 (0.49–0.82)	0.83 (0.77–0.87)	0.70 (0.62–0.78)	0.65 (0.57–0.69)	0.86 (0.80–0.89)	0.65 (0.57–0.69)	0.67 (0.55–0.74)	0.68 (0.60–0.78)	0.10 (0.07–0.13)	0.44 (0.37–0.50)	0.04 (0.01–0.06)
	≥5.0 mmol/L (80)	0.56 (0.45–0.79)	0.83 (0.75–0.86)	0.70 (0.65–0.76)	0.64 (0.55–0.70)	0.85 (0.81–0.90)	0.64 (0.55–0.70)	0.66 (0.57–0.73)	0.77 (0.62–0.90)	0.10 (0.07–0.15)	0.45 (0.38–0.53)	0.05 (0.02–0.09)
	*p*-value*	0.081	0.398	0.914	0.756	0.934	0.756	0.804	**0.003**	0.579	0.600	**0.035**
TG (374)	<1.70 mmol/L (247)	0.70 (0.49–0.81)	0.83 (0.76–0.87)	0.71 (0.63–0.78)	0.65 (0.57–0.69)	0.85 (0.80–0.89)	0.65 (0.57–0.69)	0.67 (0.56–0.74)	0.69 (0.59–0.78)	0.10 (0.07–0.14)	0.45 (0.38–0.50)	0.04 (0.02–0.07)
	≥1.70 mmol/L (127)	0.66 (0.45–0.82)	0.83 (0.76–0.87)	0.70 (0.62–0.77)	0.65 (0.57–0.70)	0.86 (0.80–0.90)	0.65 (0.57–0.70)	0.66 (0.54–0.73)	0.70 (0.60–0.83)	0.10 (0.07–0.14)	0.44 (0.34–0.51)	0.04 (0.01–0.07)
	*p*-value*	0.374	0.557	0.642	0.940	0.941	0.940	0.416	0.231	0.727	0.281	0.427
HDL (376)	<1.0 mmol/L (135)	0.63 (0.44–0.81)	0.82 (0.76–0.86)	0.70 (0.62–0.79)	0.65 (0.56–0.70)	0.86 (0.79–0.90)	0.65 (0.56–0.70)	0.67 (0.54–0.74)	0.71 (0.60–0.80)	0.09 (0.07–0.13)	0.44 (0.38–0.52)	0.04 (0.01–0.06)
	≥1.0 mmol/L (241)	0.70 (0.49–0.81)	0.83 (0.77–0.87)	0.71 (0.63–0.77)	0.64 (0.57–0.69)	0.86 (0.80–0.89)	0.64 (0.57–0.69)	0.67 (0.56–0.74)	0.68 (0.60–0.82)	0.10 (0.07–0.14)	0.45 (0.36–0.50)	0.04 (0.02–0.07)
	*p*-value*	0.102	0.204	0.860	0.951	0.955	0.951	0.599	0.511	0.185	0.374	**0.023**
LDL (376)	<3.0 mmol/L (262)	0.70 (0.49–0.82)	0.83 (0.76–0.87)	0.71 (0.61–0.78)	0.65 (0.57–0.70)	0.86 (0.80–0.89)	0.65 (0.57–0.70)	0.67 (0.55–0.74)	0.69 (0.60–0.78)	0.10 (0.07–0.13)	0.44 (0.37–0.50)	0.04 (0.01–0.06)
	≥3.0 mmol/L (114)	0.67 (0.46–0.80)	0.82 (0.76–0.87)	0.70 (0.65–0.77)	0.64 (0.55–0.70)	0.85 (0.80–0.90)	0.64 (0.55–0.70)	0.65 (0.56–0.73)	0.71 (0.59–0.87)	0.10 (0.07–0.15)	0.47 (0.38–0.53)	0.04 (0.02–0.08)
	*p*-value*	0.562	0.662	0.847	0.773	0.810	0.773	0.499	0.237	0.429	0.249	0.206

*The p-values were calculated by the Mann-Whitney test, and significant p-values are in bold.

### *F2RL3* Methylation as a Potential Biomarker for the Detection of CHD

**FIGURE 3:**
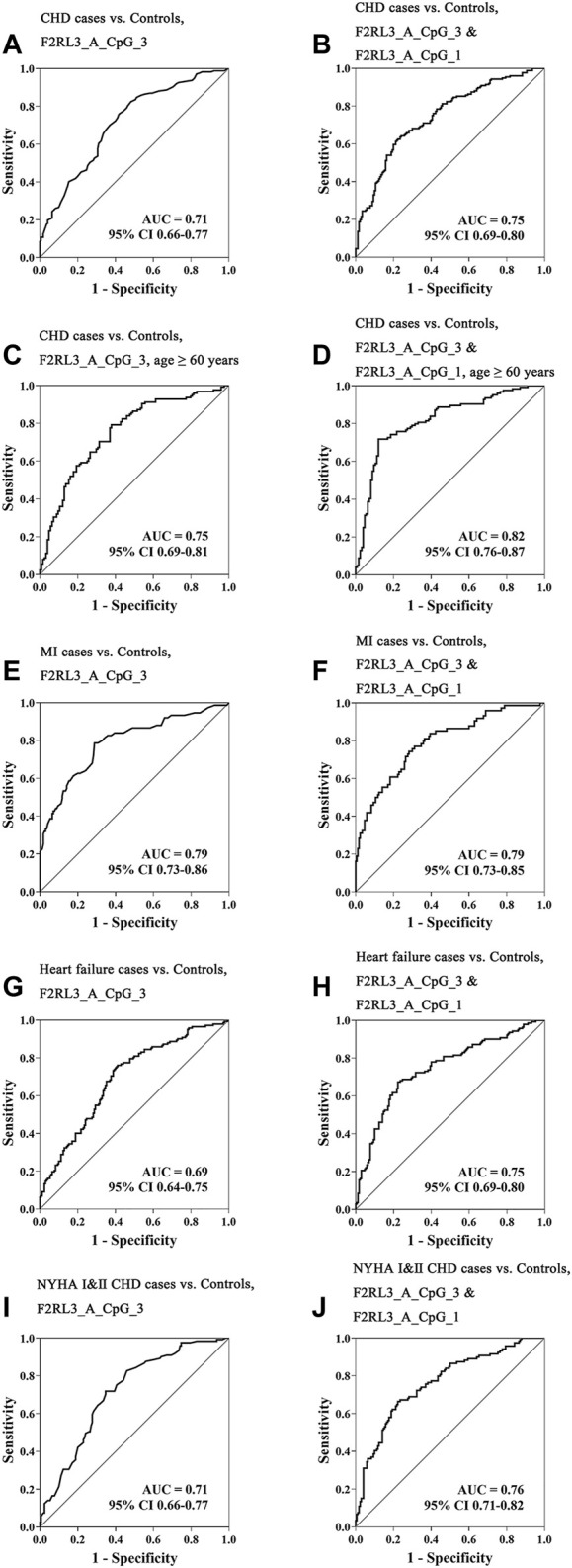
The methylation level of *F2RL3* in the peripheral blood DNA as a marker for the detection of CHD in general, age ≥60 years CHD cases, MI cases, heart failure cases, and patients with minor to medium cardiac function impairment (NYHA Ⅰ&Ⅱ CHD cases). **(A,C,E,G,I)** ROC curve analyses for the discriminatory power of F2RL3_A_CpG_3 methylation to distinguish CHD cases, age ≥60 years CHD cases, MI cases, heart failure cases, and NYHA Ⅰ&Ⅱ CHD cases from controls. **(B,D,F,H,J)** The combination of F2RL3_A_CpG_3 and F2RL3_A_CpG_1 for the discrimination of CHD cases, age ≥60 years CHD cases, MI cases, heart failure cases, and NYHA Ⅰ&Ⅱ CHD cases from controls. The ROC analyses were calculated by logistic regression adjusted for age, gender, smoking, hypertension, TC, LDL, and batch effect. The gray lines represent the line of no discrimination.

Aiming to estimate the potential clinical utility of *F2RL3* methylation as a marker for the presence of CHD, receiver operating characteristic (ROC) curve analysis was performed adjusted for age, gender, smoking, hypertension, TC levels, LDL levels, and batch effect by logistic regression. Among all the investigated 11 distinguished *F2RL3* CpG groups, F2RL3_A_CpG_3 exhibited the best discriminatory power for general CHD cases, old than 60 years CHD cases, MI cases, heart failure cases, and NYHA Ⅰ&Ⅱ CHD cases from healthy controls (area under curve (AUC) = 0.71, 0.75, 0.79, 0.69 and 0.71, respectively; Figures 3A,C,E,G,I, Supplementary Table S5). The combination of F2RL3_A_CpG_1 and F2RL3_A_CpG_3 could improve the model, and dramatically elevate the efficiency for the distinguishing of CHD cases, old than 60 years CHD cases, MI cases, heart failure cases, and NYHA Ⅰ&Ⅱ CHD cases from healthy controls (AUC = 0.75, 0.82, 0.79, 0.75 and 0.76, respectively; Figures 3B,D,F,H,J, Supplementary Table S5). However, the discriminatory power could hardly be further improved when all additional CpG sites in F2RL3_A amplicon were included in the model (Supplementary Table S5).

## Discussion

CHD is a prevalent and chronic life-threatening disease. However, there is no reliable way for early detection and risk prediction of CHD so far. DNA methylation plays a critical role in the development of cardiovascular disease with the potential to predict fundamental pathogenic processes. The previous study has demonstrated CVD mortality-related *F2RL3* (cg03636183 and cg24704287) methylation in the Caucasian population (Zhang et al., 2017). The diagnostic value of *F2RL3* methylation for CHD has not been addressed in the different ethnic populations. In the present study, we analyzed blood-based *F2RL3* methylation levels in 180 CHD patients and 184 healthy subjects in the Chinese population and proposed *F2RL3* methylation, especially methylation at CpG sites adjacent to cg03636183, as an independent biomarker for the detection of CHD controlling variant CHD-related risk factors. Moreover, we also firstly disclosed that the aberrant *F2RL3* methylation is mainly correlated with CHD in elder people, MI status, and heart failure status, and could be detected when patients have minor to medium cardiac function impairment (NYHA Ⅰ&Ⅱ CHD cases).

The *F2RL3* gene encoding for PAR-4 has been shown to play a crucial role in mediating the activation of platelet (Kahn et al., 1999; Coughlin, 2000), and multiple signaling pathways, such as immune response, the regulation of vascular endothelial cell activity, and inflammatory reactions (Vergnolle et al., 2002; Kataoka et al., 2003; Steinhoff et al., 2005). Hypomethylation is usually associated with increased gene expression, which, if this is the case with *F2RL3*, may cause increased inflammation and coagulation (Shenker et al., 2013). Several studies have reported the association between hypomethylation of *F2RL3* in the whole blood and the prognosis of CHD or increased risk of cardiovascular-related mortality, but so far there is no report about the methylation of *F2RL3* in blood and the diagnosis of CHD (Breitling et al., 2012; Zhang et al., 2014b; Zhang et al., 2017; Gao et al., 2018). In our study, we reported the association between the status of CHD and the hypomethylation of *F2RL3* in blood, more specifically at the F2RL3_A amplicon region covering cg036361837, but not at the F2RL3_B amplicon region. In addition, we also firstly observed that *F2RL3* methylation in the peripheral blood was mostly associated with CHD in people older than 60 years (especially at F2RL3_A_CpG_1; Figure 1C and Table 2C), with MI (especially at F2RL3_A_CpG_3; Figure 2A and Table 3A), heart failure patients (especially at F2RL3_A_CpG_1; Figure 2C and Table 3C) and NYHA I&II CHD patients (especially at F2RL3_A_CpG_1; Figure 2D and Table 3D). This region or CpG sites specific pattern of CHD-related *F2RL3* methylation may extend our understanding of methylation signatures.

Previous studies have revealed major differences in CHD occurring in young and old people owing to patient demographics, cardiopulmonary function, and molecular biological characteristics (Ekblom-Bak et al., 2019). As a result, the incidence of CHD is increased in people old than 65 years (Moran et al., 2008). In our study, we found that the hypomethylation of *F2RL3* is mainly associated with the risk for CHD in people above 60 years old, and this was further enhanced when people became older than 65 years old. These altered DNA methylation patterns in the blood-based *F2RL3* could be detected in minor to medium cardiac function impairment (NYHA Ⅰ&Ⅱ CHD cases) and became even more aberrant in the patients with advanced cardiac function impairment (NYHA ⅠII&IV CHD cases). Thus, our observation was consistent with the aging-related risk of CHD and suggested that the alternations of DNA methylation in blood, or say in the blood leukocytes, may play a role in the occurrence and even the progress of CHD. Nonetheless, we admitted that age can hardly be fully matched in case-control studies, and future prospective nested case-control study shall provide more robust evidence for the age-related DNA methylation alternation for the risk of CHD.

Very recently, it has been shown that blood leukocyte DNA methylation could predict the risk of future MI and CHD across diverse populations in a large-scale cohort study involving 11,461 individuals (Agha et al., 2019). Here, we specified the significant hypomethylation of *F2RL3* in the blood leukocyte DNA of MI and heart failure CHD cases compared to controls and suggested its potential clinical application as a biomarker for the prediction of MI and heart failure.

The signatures of methylation could be influenced by environmental factors and treatment (Lax and Szyf, 2018; Martin and Fry, 2018). In this study, we confirmed the strong association between the behavior of smoking and hypomethylation of *F2RL3* at cg036361837 and flanking CpG sites as reported previously (Breitling et al., 2011; Wan et al., 2012; Sun et al., 2013; Zhang et al., 2014a). But the association between hypomethylation of *F2RL3* at cg036361837 and flanking CpG sites and CHD is independent from the status of smoking since it only appeared in the non-smokers (Supplementary Table S4). We also found that the males had lower *F2RL3* methylation levels than the females whereas the hypomethylation of *F2RL3* is an indicator of high risk for CHD. This may explain why the males have a higher incidence and mortality of CHD than the females (Barrett-Connor, 2013; Benjamin et al., 2018; Ma et al., 2020). However, it seems that the blood-based *F2RL3* methylation was not influenced by drinking, hypertension, diabetes, levels of TC, TG, HDL, and LDL. Most CHD patients have a history of medication. Out of our expectations, we did not observe the obvious influence of 11 common drugs on the methylation level of *F2RL3* (Supplementary Table S6). And the correlation with digoxin should also be taken with caution since there were only 11 patients who took this drug (Supplementary Table S6). According to Breitling and the following studies (Breitling et al., 2011; Breitling et al., 2012; Zhang et al., 2014a), the hypomethylation of *F2RL3* is reversible but very slow. Even 10 years after quitting smoking, the methylation patterns of *F2RL3* in the formal smokers are still close to the smokers, and significantly differ from the non-smokers (Breitling et al., 2011; Breitling et al., 2012; Zhang et al., 2014a). Thus, *F2RL3* methylation may have a slow response to the exposure. In our study, the duration of treatment to the CHD patients is unknown, but unlikely has lasted for more than 5 years. Therefore, we can hardly observe methylation changes in the *F2RL3* gene. Whether there is drug-induced reversibility of *F2RL3* methylation, a long-term follow-up study is needed. Taken together, our intensive investigation suggested that the methylation of *F2RL3* in blood could hardly be influenced by most of the environmental factors and common medical treatment, and thus, it might be a robust and stable biomarker for the baseline initial diagnosis of CHD. Nevertheless, subjects with environmental and treatment information are still limited. The influence of CHD-related factors and medication on the *F2RL3* methylation warrants further investigations in multi-center studies with larger sample size. Moreover, the role of troponin and brain natriuretic peptide (BNP) as diagnostic biomarkers of cardiovascular disease is well established (Gaggin and Januzzi, 2013; Garg et al., 2017). However, the information of troponin and BNP are not available in the present study and should be collected in future studies. Also, the combination of *F2RL3* methylation and other types of markers, especially the markers representing different pathways or mechanisms, might provide better insight for the detection of CHD.

Differences in methylation profiles might be influenced by the proportions of the leukocyte subpopulations if cell distribution differed by disease status. However, we do not have the information of the blood cell composition in our study. Given that the altered *F2RL3* methylation was purely due to the change of leukocyte proportion in the blood, there should be a similar pattern of altered methylation in the same gene. However, we observed CHD-related methylation changes mostly in the F2RL3_A amplicon, but only slightly in the F2RL3_B amplicon. The data from the Netherlands Twin Register biobank project suggested that the interindividual differences in the cellular composition were independent of the variation observed in DNA methylation or explained only a minor proportion of this variation (Talens et al., 2010). Therefore, the observed association between *F2RL3* methylation and CHD in our study may be partly independent from the variations of blood cell composition. Moreover, there are barely reported CHD-associated mutations or common single nucleotide polymorphisms (SNPs) in the gene of *F2RL3*. Thus, we proposed that the aberrant *F2RL3* methylation in blood might be an independent risk factor for CHD. But we could not conclude if the altered *F2RL3* methylation is a causative factor or a consequence of CHD. Following studies in multi-center studies with enlarged sample size and even prospective studies are needed. Studies for the mechanism of *F2RL3* involving the circulating leukocytes would be meaningful. Unfortunately, due to the limited sample materials, we could not further explore which blood cell component plays a key role in the altered *F2RL3* methylation in blood. It is also meaningful to know if the *F2RL3* methylation contributes to the altered expression of *F2RL3* in blood. However, without fresh blood, RNA extraction is not possible in the present study. The RNA materials and the information of the blood cell proportion should be considered and collected in future studies. When possible, the *F2RL3* methylation and expression in each major cell component of cases and controls should be evaluated and compared. Meanwhile, functional studies of *F2RL3* in the cell lines and animal models would be rather helpful.

In conclusion, our study disclosed the correlation between CHD and blood-based hypomethylation of *F2RL3*, especially at cg03636183 and flanking CpG sites. This correlation appears at the early stage of cardiovascular dysfunction, and is strengthened by older age and the occurrence of MI and heart failure, but is not or just weakly influenced by most of the environmental factors and common medical treatment. We hereby suggested the blood-based *F2RL3* methylation as an objective and stable biomarker for the detection of CHD, especially for people with older age or with the status of MI. The combination of *F2RL3* methylation and conventional risk factors might be an approach to improve the risk evaluation and detection of CHD at early stage.

## Data Availability

The raw data supporting the conclusions of this article will be made available by the authors, without undue reservation, to any qualified researcher.

## References

[B1] Agha G. Mendelson M. M. Ward-Caviness C. K. Joehanes R. Huan T. Gondalia R. (2019). Blood Leukocyte DNA Methylation Predicts Risk of Future Myocardial Infarction and Coronary Heart Disease. Circulation 140 (8), 645–657. 10.1161/CIRCULATIONAHA.118.039357 31424985 PMC6812683

[B2] Arasaradnam R. P. Commane D. M. Bradburn D. Mathers J. C. (2008). A Review of Dietary Factors and its Influence on DNA Methylation in Colorectal Carcinogenesis. Epigenetics 3 (4), 193–198. 10.4161/epi.3.4.6508 18682688

[B3] Barrett-Connor E. (2013). Gender Differences and Disparities in All-Cause and Coronary Heart Disease Mortality: Epidemiological Aspects. Best Pract. Res. Clin. Endocrinol. Metab. 27 (4), 481–500. 10.1016/j.beem.2013.05.013 24054926 PMC3781943

[B4] Benjamin E. J. Virani S. S. Callaway C. W. Chamberlain A. M. Chang A. R. Cheng S. (2018). Heart Disease and Stroke Statistics-2018 Update: A Report from the American Heart Association. Circulation 137 (12), e67–e492. 10.1161/CIR.0000000000000558 29386200

[B5] Breitling L. P. Salzmann K. Rothenbacher D. Burwinkel B. Brenner H. (2012). Smoking, F2RL3 Methylation, and Prognosis in Stable Coronary Heart Disease. Eur. Heart J. 33 (22), 2841–2848. 10.1093/eurheartj/ehs091 22511653

[B6] Breitling L. P. Yang R. Korn B. Burwinkel B. Brenner H. (2011). Tobacco-Smoking-Related Differential DNA Methylation: 27K Discovery and Replication. Am. J. Hum. Genet. 88 (4), 450–457. 10.1016/j.ajhg.2011.03.003 21457905 PMC3071918

[B7] Bretschneider E. Kaufmann R. Braun M. Wittpoth M. Glusa E. Nowak G. (1999). Evidence for Proteinase-Activated Receptor-2 (PAR-2)-Mediated Mitogenesis in Coronary Artery Smooth Muscle Cells. Br. J. Pharmacol. 126 (8), 1735–1740. 10.1038/sj.bjp.0702509 10372815 PMC1565962

[B8] Coughlin S. R. (2000). Thrombin Signalling and Protease-Activated Receptors. Nature 407 (6801), 258–264. 10.1038/35025229 11001069

[B9] Danesh J. Wheeler J. G. Hirschfield G. M. Eda S. Eiriksdottir G. Rumley A. (2004). C-Reactive Protein and Other Circulating Markers of Inflammation in the Prediction of Coronary Heart Disease. N. Engl. J. Med. 350 (14), 1387–1397. 10.1056/NEJMoa032804 15070788

[B10] Ekblom-Bak E. Ekblom B. Söderling J. Börjesson M. Blom V. Kallings L. V. (2019). Sex- and Age-Specific Associations between Cardiorespiratory Fitness, CVD Morbidity and All-Cause Mortality in 266.109 Adults. Prev. Med. 127, 105799. 10.1016/j.ypmed.2019.105799 31454664

[B11] Fu H. Zhu K. Zhou D. Guan Y. Li W. Xu S. (2019). Identification and Validation of Plasma Metabolomics Reveal Potential Biomarkers for Coronary Heart Disease. Int. Heart J. 60 (6), 1387–1397. 10.1536/ihj.19-059 31666452

[B12] Gaggin H. K. Januzzi J. L. Jr (2013). Biomarkers and Diagnostics in Heart Failure. Biochim. Biophys. Acta (Bba) - Mol. Basis Dis. 1832 (12), 2442–2450. 10.1016/j.bbadis.2012.12.014 23313577

[B13] Gao B. F. Shen Z. C. Bian W. S. Wu S. X. Kang Z. X. Gao Y. (2018). Correlation of Hypertension and F2RL3 Gene Methylation with Prognosis of Coronary Heart Disease. J. Biol. Regul. Homeost Agents 32 (6), 1539–1544. 30574762

[B14] Garg P. Morris P. Fazlanie A. L. Vijayan S. Dancso B. Dastidar A. G. (2017). Cardiac Biomarkers of Acute Coronary Syndrome: from History to High-Sensitivity Cardiac Troponin. Intern. Emerg. Med. 12 (2), 147–155. 10.1007/s11739-017-1612-1 28188579 PMC5329082

[B15] Gatto L. Prati F. (2020). Subclinical Atherosclerosis: How and When to Treat it? Eur. Heart J. Suppl. 22 (Suppl. E), E87–E90. 10.1093/eurheartj/suaa068 32523447 PMC7270961

[B16] Hansson G. K. (2005). Inflammation, Atherosclerosis, and Coronary Artery Disease. N. Engl. J. Med. 352 (16), 1685–1695. 10.1056/NEJMra043430 15843671

[B17] Horvath S. Raj K. (2018). DNA Methylation-Based Biomarkers and the Epigenetic Clock Theory of Ageing. Nat. Rev. Genet. 19 (6), 371–384. 10.1038/s41576-018-0004-3 29643443

[B18] Jellinger P. S. Handelsman Y. Rosenblit P. D. Bloomgarden Z. T. Fonseca V. A. Garber A. J. (2017). American Association of Clinical Endocrinologists and American College of Endocrinology Guidelines for Management of Dyslipidemia and Prevention of Cardiovascular Disease. Endocr. Pract. 23 (Suppl. 2), 1–87. 10.4158/EP171764.APPGL 28437620

[B19] Jia L. Zhu L. Wang J. Z. Wang X. J. Chen J. Z. Song L. (2013). Methylation of FOXP3 in Regulatory T Cells Is Related to the Severity of Coronary Artery Disease. Atherosclerosis 228 (2), 346–352. 10.1016/j.atherosclerosis.2013.01.027 23566804

[B20] Kahn M. L. Nakanishi-Matsui M. Shapiro M. J. Ishihara H. Coughlin S. R. (1999). Protease-Activated Receptors 1 and 4 Mediate Activation of Human Platelets by Thrombin. J. Clin. Invest. 103 (6), 879–887. 10.1172/JCI6042 10079109 PMC408153

[B21] Kataoka H. Hamilton J. R. McKemy D. D. Camerer E. Zheng Y.-W. Cheng A. (2003). Protease-Activated Receptors 1 and 4 Mediate Thrombin Signaling in Endothelial Cells. Blood 102 (9), 3224–3231. 10.1182/blood-2003-04-1130 12869501

[B22] Kolpakov M. A. Rafiq K. Guo X. Hooshdaran B. Wang T. Vlasenko L. (2016). Protease-Activated Receptor 4 Deficiency Offers Cardioprotection after Acute Ischemia Reperfusion Injury. J. Mol. Cell Cardiol. 90, 21–29. 10.1016/j.yjmcc.2015.11.030 26643815 PMC5332160

[B23] Laird P. W. (2003). The Power and the Promise of DNA Methylation Markers. Nat. Rev. Cancer 3 (4), 253–266. 10.1038/nrc1045 12671664

[B24] Lax E. Szyf M. (2018). The Role of DNA Methylation in Drug Addiction: Implications for Diagnostic and Therapeutics. Prog. Mol. Biol. Transl Sci. 157, 93–104. 10.1016/bs.pmbts.2018.01.003 29933958

[B25] Leger A. J. Covic L. Kuliopulos A. (2006). Protease-Activated Receptors in Cardiovascular Diseases. Circulation 114 (10), 1070–1077. 10.1161/CIRCULATIONAHA.105.574830 16952995

[B26] Li H. Sun K. Zhao R. Hu J. Hao Z. Wang F. (2018). Inflammatory Biomarkers of Coronary Heart Disease. Front. Biosci. (Schol Ed) 10, 185–196. 10.2741/s508 28930526

[B27] Lobbes M. B. Kooi M. E. Lutgens E. Ruiters A. W. Lima Passos V. Braat S. H. (2010). Leukocyte Counts, Myeloperoxidase, and Pregnancy-Associated Plasma Protein a as Biomarkers for Cardiovascular Disease: Towards a Multi-Biomarker Approach. Int. J. Vasc. Med. 2010, 726207. 10.1155/2010/726207 21188207 PMC3003971

[B28] Ma L. Y. Chen W. W. Gao R. L. Liu L. S. Zhu M. L. Wang Y. J. (2020). China Cardiovascular Diseases Report 2018: An Updated Summary. J. Geriatr. Cardiol. 17 (1), 1–8. 10.11909/j.issn.1671-5411.2020.01.001 32133031 PMC7008101

[B29] Martin E. M. Fry R. C. (2018). Environmental Influences on the Epigenome: Exposure- Associated DNA Methylation in Human Populations. Annu. Rev. Public Health 39, 309–333. 10.1146/annurev-publhealth-040617-014629 29328878

[B30] Moran A. Zhao D. Gu D. Coxson P. Chen C.-S. Cheng J. (2008). The Future Impact of Population Growth and Aging on Coronary Heart Disease in China: Projections from the Coronary Heart Disease Policy Model-China. BMC Public Health 8, 394. 10.1186/1471-2458-8-394 19036167 PMC2631484

[B31] Peng P. Wang L. Yang X. Huang X. Ba Y. Chen X. (2014). A Preliminary Study of the Relationship between Promoter Methylation of the ABCG1, GALNT2 and HMGCR Genes and Coronary Heart Disease. PLoS One 9 (8), e102265. 10.1371/journal.pone.0102265 25084356 PMC4118847

[B32] Robertson K. D. Wolffe A. P. (2000). DNA Methylation in Health and Disease. Nat. Rev. Genet. 1 (1), 11–19. 10.1038/35049533 11262868

[B33] Schleithoff C. Voelter-Mahlknecht S. Dahmke I. N. Mahlknecht U. (2012). On the Epigenetics of Vascular Regulation and Disease. Clin. Epigenet 4 (1), 7. 10.1186/1868-7083-4-7 PMC3438017 22621747

[B34] Shaya G. E. Leucker T. M. Jones S. R. Martin S. S. Toth P. P. (2021). Coronary Heart Disease Risk: Low-Density Lipoprotein and Beyond. Trends Cardiovasc. Med. S1050-1738 (21), 00046–53. 10.1016/j.tcm.2021.04.002 33872757

[B35] Shenker N. S. Polidoro S. van Veldhoven K. Sacerdote C. Ricceri F. Birrell M. A. (2013). Epigenome-Wide Association Study in the European Prospective Investigation into Cancer and Nutrition (EPIC-Turin) Identifies Novel Genetic Loci Associated with Smoking. Hum. Mol. Genet. 22 (5), 843–851. 10.1093/hmg/dds488 23175441

[B36] Steinhoff M. Buddenkotte J. Shpacovitch V. Rattenholl A. Moormann C. Vergnolle N. (2005). Proteinase-Activated Receptors: Transducers of Proteinase-Mediated Signaling in Inflammation and Immune Response. Endocr. Rev. 26 (1), 1–43. 10.1210/er.2003-0025 15689571

[B37] Sun Y. V. Smith A. K. Conneely K. N. Chang Q. Li W. Lazarus A. (2013). Epigenomic Association Analysis Identifies Smoking-Related DNA Methylation Sites in African Americans. Hum. Genet. 132 (9), 1027–1037. 10.1007/s00439-013-1311-6 23657504 PMC3744600

[B38] Talens R. P. Boomsma D. I. Tobi E. W. Kremer D. Jukema J. W. Willemsen G. (2010). Variation, Patterns, and Temporal Stability of DNA Methylation: Considerations for Epigenetic Epidemiology. FASEB j. 24 (9), 3135–3144. 10.1096/fj.09-150490 20385621

[B39] Talmud P. J. (2007). Gene-Environment Interaction and its Impact on Coronary Heart Disease Risk. Nutr. Metab. Cardiovasc. Dis. 17 (2), 148–152. 10.1016/j.numecd.2006.01.008 17306734

[B40] Vergnolle N. Derian C. K. D’Andrea M. R. Steinhoff M. Andrade-Gordon P. (2002). Characterization of Thrombin-Induced Leukocyte Rolling and Adherence: A Potential Proinflammatory Role for Proteinase-Activated Receptor-4. J. Immunol. 169 (3), 1467–1473. 10.4049/jimmunol.169.3.1467 12133973

[B41] Virani S. S. Alonso A. Aparicio H. J. Benjamin E. J. Bittencourt M. S. Callaway C. W. (2021). Heart Disease and Stroke Statistics-2021 Update: A Report from the American Heart Association. Circulation 143 (8), e254–e743. 10.1161/CIR.0000000000000950 33501848 PMC13036842

[B42] Vorchheimer D. A. Becker R. (2006). Platelets in Atherothrombosis. Mayo Clinic Proc. 81 (1), 59–68. 10.4065/81.1.59 16438480

[B43] Wan E. S. Qiu W. Baccarelli A. Carey V. J. Bacherman H. Rennard S. I. (2012). Cigarette Smoking Behaviors and Time since Quitting Are Associated with Differential DNA Methylation across the Human Genome. Hum. Mol. Genet. 21 (13), 3073–3082. 10.1093/hmg/dds135 22492999 PMC3373248

[B44] Wang J. Tan G. J. Han L. N. Bai Y. Y. He M. Liu H. B. (2017). Novel Biomarkers for Cardiovascular Risk Prediction. J. Geriatr. Cardiol. 14 (2), 135–150. 10.11909/j.issn.1671-5411.2017.02.008 28491088 PMC5409355

[B45] Weber M. Davies J. J. Wittig D. Oakeley E. J. Haase M. Lam W. L. (2005). Chromosome-Wide and Promoter-Specific Analyses Identify Sites of Differential DNA Methylation in normal and Transformed Human Cells. Nat. Genet. 37 (8), 853–862. 10.1038/ng1598 16007088

[B46] Wei M. Liu Y. Zheng M. Wang L. Ma F. Qi Y. (2019). Upregulation of Protease-Activated Receptor 2 Promotes Proliferation and Migration of Human Vascular Smooth Muscle Cells (VSMCs). Med. Sci. Monit. 25, 8854–8862. 10.12659/MSM.917865 31756174 PMC6883764

[B47] Yancy C. W. Jessup M. Bozkurt B. Butler J. Casey D. E. Jr. Colvin M. M. (2017). 2017 ACC/AHA/HFSA Focused Update of the 2013 ACCF/AHA Guideline for the Management of Heart Failure: A Report of the American College of Cardiology/American Heart Association Task Force on Clinical Practice Guidelines and the Heart Failure Society of America. J. Card. Fail. 23 (8), 628–651. 10.1016/j.cardfail.2017.04.014 28461259

[B48] Yang R. Pfütze K. Zucknick M. Sutter C. Wappenschmidt B. Marme F. (2015). DNA Methylation Array Analyses Identified Breast Cancer-AssociatedHYAL2methylation in Peripheral Blood. Int. J. Cancer 136 (8), 1845–1855. 10.1002/ijc.29205 25213452

[B49] Zhang L. Zhang Y. Zhao Y. Wang Y. Ding H. Xue S. (2018). Circulating miRNAs as Biomarkers for Early Diagnosis of Coronary Artery Disease. Expert Opin. Ther. Patents 28 (8), 591–601. 10.1080/13543776.2018.1503650 30064285

[B50] Zhang Y. Wilson R. Heiss J. Breitling L. P. Saum K.-U. Schöttker B. (2017). DNA Methylation Signatures in Peripheral Blood Strongly Predict All-Cause Mortality. Nat. Commun. 8, 14617. 10.1038/ncomms14617 28303888 PMC5357865

[B51] Zhang Y. Yang R. Burwinkel B. Breitling L. P. Brenner H. (2014a). F2RL3 Methylation as a Biomarker of Current and Lifetime Smoking Exposures. Environ. Health Perspect. 122 (2), 131–137. 10.1289/ehp.1306937 24273234 PMC3915264

[B52] Zhang Y. Yang R. Burwinkel B. Breitling L. P. Holleczek B. Schöttker B. (2014b). F2RL3methylation in Blood DNA Is a strong Predictor of Mortality. Int. J. Epidemiol. 43 (4), 1215–1225. 10.1093/ije/dyu006 24510982 PMC4258765

[B53] Zuo H. P. Guo Y. Y. Che L. Wu X. Z. (2016). Hypomethylation of Interleukin-6 Promoter Is Associated with the Risk of Coronary Heart Disease. Arq Bras Cardiol. 107 (2), 131–136. 10.5935/abc.20160124 27627640 PMC5074066

