# Supplementary material for: F2RL3 Methylation in the Peripheral Blood as a Potential Marker for the Detection of Coronary Heart Disease: A Case-Control Study
Source: Front Genet. 2022 Mar 24;13:833923. doi: 10.3389/fgene.2022.833923 (PMC8996303; doi:10.3389/fgene.2022.833923)
Supplement: Supplementary file 1 [file DataSheet1.doc]

**SUPPLEMENTAL MATERIAL**

**Title:** *F2RL3* methylation in the peripheral blood as a potential marker for the detection of [coronary heart disease](http://www.baidu.com/link?url=BjTnQxOUyMa23gY-kV8Amz0OQ3GmdDJbQnWekDeUCsUCRyxBnNhCsmkGz_nN3phTzZ-g0ixpTiHihJ4wHCXSjZqaiqXAU1B0hIwNFasp69U5JSDoQxV9WKwWdcqUMHOM): a case-control study

**Running Title:** DNA Methylation in CHD

**Xiaojing Zhao^1,2†^, Liya Zhu^3†^, Qiming Yin^3^, Zhenguo Xu^4,5^, Qian Jia^1,2^,** [**Rongxi Yang**](http://loop.frontiersin.org/people/974610/overview)**^3*^, Kunlun He^1,2*^**

^1^Military translational medicine lab, Medical Innovation Research Division, Chinese PLA General Hospital, Beijing, China

^2^Beijing Key Laboratory of Chronic Heart Failure Precision Medicine, Medical Innovation Research Division, Chinese PLA General Hospital, Beijing, China

^3^Department of Epidemiology and Biostatistics, School of Public Health, Nanjing Medical University, Nanjing, China

^4^The First Medical Center, Chinese PLA General Hospital, Beijing, China

^5^The medical school of Chinese PLA, Beijing, China

*** Correspondence:**

Rongxi Yang

[rongxiyang@njmu.edu.cn](mailto:rongxiyang@njmu.edu.cn)

Kunlun He

[kunlunhe@plagh.org](mailto:kunlunhe@plagh.org)

^†^These authors have contributed equally to this work and share first authorship

**Supplementary Tables**

**Supplementary Table 1. Age-related methylation difference of *F2RL3* in CHD cases and controls**

| **A. Age < 65 years** | | | | | | | | | | |
| --- | --- | --- | --- | --- | --- | --- | --- | --- | --- | --- |
| **CpG sites** | **Controls (N=119)** | **CHD cases (N=83)** | **Model 1 ^a^** | |  | **Model 2 ^b^** | |  | **Model 3 ^c^** | |
|  | **Median (IQR)** | **Median (IQR)** | **OR (95%CI) per-10% methylation** | ***p-*value** |  | **OR (95%CI) per-10% methylation** | ***p-*value** |  | **OR (95%CI) per-10% methylation** | ***p-*value** |
| F2RL3_A_CpG_1 | 0.68(0.48-0.81) | 0.65(0.47-0.80) | 1.04(0.90-1.20) | 0.624 |  | 1.03(0.89-1.19) | 0.727 |  | 1.07(0.90-1.27) | 0.437 |
| F2RL3_A_CpG_2/cg03636183 | 0.83(0.78-0.87) | 0.83(0.76-0.87) | 1.14(0.85-1.54) | 0.378 |  | 1.16(0.85-1.59) | 0.347 |  | 1.15(0.80-1.64) | 0.448 |
| F2RL3_A_CpG_3 | 0.72(0.66-0.77) | 0.72(0.53-0.80) | 1.33(1.08-1.64) | **0.008** |  | 1.28(1.03-1.59) | **0.026** |  | 1.34(1.03-1.73) | **0.027** |
| F2RL3_A_CpG_4 | 0.66(0.59-0.71) | 0.63(0.57-0.70) | 1.27(0.97-1.66) | 0.080 |  | 1.26(0.94-1.68) | 0.128 |  | 1.31(0.94-1.83) | 0.115 |
| F2RL3_A_CpG_5 | 0.86(0.82-0.89) | 0.85(0.77-0.90) | 1.37(1.00-1.88) | 0.051 |  | 1.41(1.01-1.97) | **0.046** |  | 1.46(0.99-2.15) | 0.055 |
| F2RL3_A_CpG_6 | 0.66(0.59-0.71) | 0.63(0.57-0.70) | 1.27(0.97-1.66) | 0.080 |  | 1.26(0.94-1.68) | 0.128 |  | 1.31(0.94-1.83) | 0.115 |
| F2RL3_A_CpG_7 | 0.69(0.59-0.76) | 0.67(0.53-0.73) | 1.23(0.99-1.53) | 0.067 |  | 1.19(0.94-1.51) | 0.150 |  | 1.15(0.88-1.51) | 0.301 |
| F2RL3_B_CpG_2 | 0.70(0.58-0.82) | 0.72(0.65-0.85) | 0.86(0.73-1.02) | 0.078 |  | 0.88(0.75-1.04) | 0.142 |  | 0.86(0.70-1.06) | 0.147 |
| F2RL3_B_CpG_4.5 | 0.10(0.07-0.13) | 0.10(0.07-0.14) | 1.06(0.75-1.51) | 0.735 |  | 1.10(0.77-1.58) | 0.594 |  | 1.12(0.76-1.64) | 0.562 |
| F2RL3_B_CpG_6 | 0.45(0.39-0.52) | 0.45(0.38-0.51) | 1.01(0.82-1.25) | 0.916 |  | 0.99(0.79-1.22) | 0.895 |  | 1.03(0.80-1.33) | 0.826 |
| F2RL3_B_CpG_7 | 0.04(0.02-0.07) | 0.04(0.01-0.06) | 1.93(0.91-4.09) | 0.084 |  | 1.75(0.83-3.70) | 0.144 |  | 1.63(0.85-3.13) | 0.142 |
| **B. Age ≥ 65 years** | | | | | | | | | | |
| **CpG sites** | **Controls (N=65)** | **CHD cases (N=97)** | **Model 1 ^a^** | |  | **Model 2 ^b^** | |  | **Model 3 ^c^** | |
|  | **Median (IQR)** | **Median (IQR)** | **OR (95%CI) per-10% methylation** | ***p-*value** |  | **OR (95%CI) per-10% methylation** | ***p-*value** |  | **OR (95%CI) per-10% methylation** | ***p-*value** |
| F2RL3_A_CpG_1 | 0.77(0.53-0.85) | 0.61(0.43-0.78) | 1.24(1.06-1.45) | **0.007** |  | 1.23(1.05-1.45) | **0.010** |  | 1.48(1.19-1.84) | **4.40E-04** |
| F2RL3_A_CpG_2/cg03636183 | 0.84(0.79-0.87) | 0.82(0.74-0.86) | 1.35(0.94-1.94) | 0.100 |  | 1.43(0.99-2.08) | 0.057 |  | 1.75(1.12-2.72) | **0.013** |
| F2RL3_A_CpG_3 | 0.70(0.66-0.78) | 0.70(0.56-0.79) | 1.27(1.01-1.61) | **0.046** |  | 1.35(1.06-1.72) | **0.015** |  | 1.43(1.09-1.89) | **0.010** |
| F2RL3_A_CpG_4 | 0.65(0.58-0.70) | 0.62(0.53-0.68) | 1.40(1.01-1.95) | **0.044** |  | 1.45(1.03-2.04) | **0.032** |  | 1.55(1.05-2.28) | **0.027** |
| F2RL3_A_CpG_5 | 0.85(0.81-0.89) | 0.85(0.76-0.89) | 1.10(0.81-1.50) | 0.549 |  | 1.12(0.81-1.55) | 0.486 |  | 1.12(0.79-1.58) | 0.521 |
| F2RL3_A_CpG_6 | 0.65(0.58-0.70) | 0.62(0.53-0.68) | 1.40(1.01-1.95) | **0.044** |  | 1.45(1.03-2.04) | **0.032** |  | 1.55(1.05-2.28) | **0.027** |
| F2RL3_A_CpG_7 | 0.68(0.57-0.74) | 0.65(0.51-0.72) | 1.25(0.97-1.61) | 0.090 |  | 1.26(0.97-1.64) | 0.085 |  | 1.32(0.98-1.79) | 0.068 |
| F2RL3_B_CpG_2 | 0.62(0.56-0.77) | 0.69(0.62-0.77) | 0.87(0.71-1.05) | 0.138 |  | 0.88(0.72-1.07) | 0.184 |  | 0.92(0.73-1.16) | 0.477 |
| F2RL3_B_CpG_4.5 | 0.10(0.08-0.14) | 0.09(0.06-0.12) | 1.82(1.00-3.30) | 0.050 |  | 1.67(0.91-3.07) | 0.098 |  | 1.60(0.82-3.13) | 0.172 |
| F2RL3_B_CpG_6 | 0.45(0.40-0.49) | 0.41(0.31-0.51) | 1.22(0.92-1.61) | 0.168 |  | 1.19(0.90-1.59) | 0.221 |  | 1.21(0.88-1.66) | 0.233 |
| F2RL3_B_CpG_7 | 0.04(0.03-0.07) | 0.04(0.01-0.06) | 2.06(0.86-4.95) | 0.107 |  | 1.81(0.74-4.40) | 0.191 |  | 1.52(0.54-4.28) | 0.432 |

^a^ Model 1: Logistic regression without adjustment; ^b^ Model 2: Logistic regression adjusted for age and gender; ^c^ Model 3: Logistic regression adjusted for age, gender, smoking, hypertension, total cholesterol (TC), low density lipoprotein (LDL), and batch effect. Significant *p*-values are in bold.

**Supplementary Table 2**. Methylation difference of *F2RL3* comparing NYHA Ⅲ&Ⅳ CHD cases and controls

| **CpG sites** | **Controls (N=184)** | **NYHA Ⅲ&Ⅳ CHD cases (N=37)** | **Model 1 ^a^** | |  | **Model 2 ^b^** | |  | **Model 3 ^c^** | |
| --- | --- | --- | --- | --- | --- | --- | --- | --- | --- | --- |
|  | **Median (IQR)** | **Median (IQR)** | **OR (95%CI) per-10% methylation** | ***p-*value** |  | **OR (95%CI) per-10% methylation** | ***p-*value** |  | **OR (95%CI) per-10% methylation** | ***p-*value** |
| F2RL3_A_CpG_1 | 0.71(0.49-0.82) | 0.72(0.49-0.80) | 1.05(0.88-1.25) | 0.611 |  | 1.06(0.89-1.27) | 0.488 |  | 1.16(0.95-1.42) | 0.157 |
| F2RL3_A_CpG_2/cg03636183 | 0.83(0.79-0.87) | 0.83(0.71-0.87) | 1.50(1.05-2.14) | **0.026** |  | 1.47(1.02-2.11) | **0.039** |  | 1.48(0.99-2.22) | 0.055 |
| F2RL3_A_CpG_3 | 0.71(0.66-0.77) | 0.65(0.45-0.77) | 1.92(1.41-2.61) | **3.60E-05** |  | 2.01(1.46-2.78) | **2.20E-05** |  | 1.99(1.42-2.78) | **6.30E-05** |
| F2RL3_A_CpG_4 | 0.66(0.59-0.70) | 0.60(0.48-0.68) | 1.62(1.17-2.22) | **0.003** |  | 1.53(1.10-2.13) | **0.012** |  | 1.54(1.07-2.21) | **0.020** |
| F2RL3_A_CpG_5 | 0.86(0.82-0.89) | 0.81(0.73-0.88) | 1.49(1.07-2.06) | **0.017** |  | 1.41(1.00-1.98) | **0.049** |  | 1.35(0.96-1.90) | 0.089 |
| F2RL3_A_CpG_6 | 0.66(0.59-0.70) | 0.60(0.48-0.68) | 1.62(1.17-2.22) | **0.003** |  | 1.53(1.10-2.13) | **0.012** |  | 1.54(1.07-2.21) | **0.020** |
| F2RL3_A_CpG_7 | 0.69(0.58-0.75) | 0.63(0.46-0.69) | 1.57(1.20-2.07) | **0.001** |  | 1.51(1.13-2.00) | **0.005** |  | 1.49(1.10-2.02) | **0.010** |
| F2RL3_B_CpG_2 | 0.67(0.57-0.81) | 0.69(0.62-0.77) | 0.97(0.79-1.19) | 0.746 |  | 0.94(0.76-1.17) | 0.569 |  | 0.94(0.74-1.20) | 0.613 |
| F2RL3_B_CpG_4.5 | 0.10(0.08-0.14) | 0.08(0.05-0.11) | 2.91(1.22-6.95) | **0.016** |  | 2.54(1.04-6.21) | **0.041** |  | 2.36(0.88-6.33) | 0.088 |
| F2RL3_B_CpG_6 | 0.45(0.39-0.50) | 0.41(0.30-0.52) | 1.09(0.80-1.49) | 0.573 |  | 1.02(0.74-1.40) | 0.916 |  | 1.04(0.76-1.44) | 0.793 |
| F2RL3_B_CpG_7 | 0.04(0.02-0.07) | 0.03(0.01-0.07) | 1.94(0.71-5.29) | 0.196 |  | 1.82(0.64-5.20) | 0.261 |  | 2.05(0.66-6.36) | 0.213 |

^a^ Model 1: Logistic regression without adjustment; ^b^ Model 2: Logistic regression adjusted for age and gender; ^c^ Model 3: Logistic regression adjusted for age, gender, smoking, hypertension, TC, LDL, and batch effect. Significant *p*-values are in bold. NYHA, New York Heart Association.

**Supplementary Table 3.** NYHA classification and the methylation intensity of *F2RL3*

| **CpG sites** | **NYHA Ⅰ&Ⅱ CHD cases (N=124)** | **NYHA Ⅲ&Ⅳ CHD cases (N=37)** | ***p-*value *** |
| --- | --- | --- | --- |
|  | **Median (IQR)** | **Median (IQR)** |  |
| F2RL3_A_CpG_1 | 0.56(0.40-0.78) | 0.72(0.49-0.80) | 0.114 |
| F2RL3_A_CpG_2/cg03636183 | 0.82(0.77-0.87) | 0.83(0.71-0.87) | 0.498 |
| F2RL3_A_CpG_3 | 0.73(0.61-0.82) | 0.65(0.45-0.77) | **0.031** |
| F2RL3_A_CpG_4 | 0.63(0.57-0.68) | 0.60(0.48-0.68) | 0.203 |
| F2RL3_A_CpG_5 | 0.87(0.80-0.90) | 0.81(0.73-0.88) | **0.025** |
| F2RL3_A_CpG_6 | 0.63(0.57-0.68) | 0.60(0.48-0.68) | 0.203 |
| F2RL3_A_CpG_7 | 0.66(0.53-0.72) | 0.63(0.46-0.69) | 0.125 |
| F2RL3_B_CpG_2 | 0.71(0.62-0.81) | 0.69(0.62-0.77) | 0.373 |
| F2RL3_B_CpG_4.5 | 0.10(0.07-0.14) | 0.08(0.05-0.11) | **0.032** |
| F2RL3_B_CpG_6 | 0.45(0.39-0.51) | 0.41(0.30-0.52) | 0.304 |
| F2RL3_B_CpG_7 | 0.04(0.01-0.06) | 0.03(0.01-0.07) | 0.775 |

^*^ The *p*-values were calculated by the Mann-Whitney test, and significant *p*-values are in bold. NYHA, New York Heart Association.

**Supplementary Table 4.** Association between CHD and *F2RL3* methylation stratified by the status of smoking

| **Non-smoker** | | | | | | | | | | |
| --- | --- | --- | --- | --- | --- | --- | --- | --- | --- | --- |
| **CpG sites** | **Controls (N=127)** | **CHD cases (N=107)** | **Model 1 ^a^** | |  | **Model 2 ^b^** | |  | **Model 3 ^c^** | |
|  | **Median (IQR)** | **Median (IQR)** | **OR (95%CI) per-10% methylation** | ***p-*value** |  | **OR (95%CI) per-10% methylation** | ***p-*value** |  | **OR (95%CI) per-10% methylation** | ***p-*value** |
| F2RL3_A_CpG_1 | 0.73(0.51-0.83) | 0.66(0.47-0.79) | 1.13(0.99-1.29) | 0.069 |  | 1.13(0.99-1.30) | 0.067 |  | 1.28(1.09-1.51) | **0.002** |
| F2RL3_A_CpG_2/cg03636183 | 0.84(0.80-0.87) | 0.83(0.78-0.87) | 1.36(0.99-1.86) | 0.059 |  | 1.38(1.00-1.90) | 0.053 |  | 1.59(1.11-2.29) | **0.012** |
| F2RL3_A_CpG_3 | 0.71(0.66-0.77) | 0.71(0.54-0.78) | 1.34(1.10-1.64) | **0.004** |  | 1.40(1.14-1.72) | **0.001** |  | 1.42(1.13-1.78) | **0.002** |
| F2RL3_A_CpG_4 | 0.67(0.63-0.71) | 0.66(0.60-0.69) | 1.39(1.04-1.85) | **0.026** |  | 1.36(1.01-1.81) | **0.040** |  | 1.47(1.07-2.02) | **0.017** |
| F2RL3_A_CpG_5 | 0.87(0.84-0.90) | 0.86(0.80-0.91) | 1.14(0.87-1.49) | 0.331 |  | 1.12(0.85-1.48) | 0.415 |  | 1.14(0.84-1.55) | 0.399 |
| F2RL3_A_CpG_6 | 0.67(0.63-0.71) | 0.66(0.60-0.69) | 1.39(1.04-1.85) | **0.026** |  | 1.36(1.01-1.81) | **0.040** |  | 1.47(1.07-2.02) | **0.017** |
| F2RL3_A_CpG_7 | 0.72(0.63-0.76) | 0.68(0.60-0.74) | 1.29(1.03-1.62) | **0.030** |  | 1.26(0.99-1.59) | 0.057 |  | 1.35(1.05-1.74) | **0.021** |
| F2RL3_B_CpG_2 | 0.65(0.56-0.80) | 0.72(0.64-0.81) | 0.90(0.77-1.04) | 0.158 |  | 0.87(0.75-1.02) | 0.080 |  | 0.89(0.75-1.06) | 0.204 |
| F2RL3_B_CpG_4.5 | 0.11(0.08-0.14) | 0.10(0.07-0.14) | 1.23(0.77-1.96) | 0.390 |  | 1.19(0.74-1.92) | 0.477 |  | 1.00(0.61-1.66) | 0.991 |
| F2RL3_B_CpG_6 | 0.44(0.39-0.49) | 0.45(0.37-0.52) | 1.03(0.82-1.29) | 0.792 |  | 1.02(0.81-1.29) | 0.842 |  | 0.97(0.76-1.24) | 0.806 |
| F2RL3_B_CpG_7 | 0.04(0.02-0.07) | 0.04(0.01-0.06) | 1.92(0.97-3.78) | 0.060 |  | 1.91(0.96-3.77) | 0.065 |  | 1.75(0.84-3.66) | 0.137 |
| **Smoker** | | | | | | | | | | |
| **CpG sites** | **Controls (N=53)** | **CHD cases (N=73)** | **Model 1 ^a^** | |  | **Model 2 ^b^** | |  | **Model 3 ^c^** | |
|  | **Median (IQR)** | **Median (IQR)** | **OR (95%CI) per-10% methylation** | ***p-*value** |  | **OR (95%CI) per-10% methylation** | ***p-*value** |  | **OR (95%CI) per-10% methylation** | ***p-*value** |
| F2RL3_A_CpG_1 | 0.71(0.46-0.81) | 0.60(0.40-0.78) | 1.11(0.94-1.31) | 0.212 |  | 1.11(0.94-1.32) | 0.215 |  | 1.16(0.95-1.41) | 0.143 |
| F2RL3_A_CpG_2/cg03636183 | 0.80(0.72-0.84) | 0.78(0.72-0.86) | 1.00(0.70-1.41) | 0.983 |  | 0.99(0.69-1.41) | 0.945 |  | 1.04(0.71-1.52) | 0.845 |
| F2RL3_A_CpG_3 | 0.69(0.65-0.78) | 0.70(0.55-0.80) | 1.24(0.96-1.59) | 0.101 |  | 1.27(0.99-1.64) | 0.064 |  | 1.42(1.05-1.91) | **0.021** |
| F2RL3_A_CpG_4 | 0.61(0.52-0.67) | 0.57(0.49-0.65) | 1.22(0.87-1.70) | 0.254 |  | 1.24(0.88-1.74) | 0.222 |  | 1.39(0.95-2.04) | 0.088 |
| F2RL3_A_CpG_5 | 0.82(0.78-0.88) | 0.80(0.72-0.88) | 1.37(0.91-2.07) | 0.127 |  | 1.39(0.92-2.11) | 0.119 |  | 1.41(0.90-2.22) | 0.131 |
| F2RL3_A_CpG_6 | 0.61(0.52-0.67) | 0.57(0.49-0.65) | 1.22(0.87-1.70) | 0.254 |  | 1.24(0.88-1.74) | 0.222 |  | 1.39(0.95-2.04) | 0.088 |
| F2RL3_A_CpG_7 | 0.60(0.50-0.70) | 0.56(0.47-0.69) | 1.12(0.86-1.46) | 0.420 |  | 1.14(0.87-1.50) | 0.341 |  | 1.22(0.90-1.64) | 0.194 |
| F2RL3_B_CpG_2 | 0.67(0.59-0.80) | 0.71(0.62-0.83) | 0.85(0.69-1.05) | 0.123 |  | 0.85(0.69-1.05) | 0.135 |  | 0.80(0.62-1.03) | 0.079 |
| F2RL3_B_CpG_4.5 | 0.09(0.08-0.13) | 0.09(0.05-0.13) | 1.22(0.79-1.90) | 0.375 |  | 1.20(0.77-1.89) | 0.418 |  | 1.28(0.81-2.02) | 0.291 |
| F2RL3_B_CpG_6 | 0.47(0.40-0.52) | 0.43(0.30-0.51) | 1.21(0.94-1.57) | 0.139 |  | 1.21(0.93-1.57) | 0.153 |  | 1.28(0.97-1.69) | 0.087 |
| F2RL3_B_CpG_7 | 0.04(0.03-0.07) | 0.03(0.01-0.06) | 1.98(0.71-5.52) | 0.190 |  | 2.21(0.76-6.47) | 0.146 |  | 2.73(0.83-8.99) | 0.099 |

^a^ Model 1: Logistic regression without adjustment; ^b^ Model 2: Logistic regression adjusted for age and gender; ^c^ Model 3: Logistic regression adjusted for age, gender, hypertension, TC, LDL, and batch effect. Significant *p*-values are in bold.

**Supplementary Table 5.** The discriminatory power of *F2RL3* methylation to distinguish CHD cases from controls

| **All 180 CHD cases vs. all 184 controls** | | | |
| --- | --- | --- | --- |
| **CpG sites** | **AUC, 95% CI ^a^** |  | **AUC, 95% CI ^b^** |
| F2RL3_A_CpG_3 | 0.63(0.57-0.69) |  | 0.71(0.66-0.77) |
| F2RL3_A_CpG_1 & F2RL3_A_CpG_3 | 0.66(0.61-0.72) |  | 0.75(0.69-0.80) |
| All seven F2RL3_A_CpGs | 0.66(0.61-0.72) |  | 0.75(0.70-0.80) |
| **127 CHD cases vs. 133 controls, age ≥ 60 years** | | | |
| **CpG sites** | **AUC, 95% CI ^a^** |  | **AUC, 95% CI ^b^** |
| F2RL3_A_CpG_3 | 0.71(0.64-0.77) |  | 0.75(0.69-0.81) |
| F2RL3_A_CpG_1 & F2RL3_A_CpG_3 | 0.75(0.70-0.81) |  | 0.82(0.76-0.87) |
| All seven F2RL3_A_CpGs | 0.76(0.70-0.81) |  | 0.82(0.76-0.87) |
| **78 MI cases vs. all 184 controls** | | | |
| **CpG sites** | **AUC, 95% CI ^a^** |  | **AUC, 95% CI ^b^** |
| F2RL3_A_CpG_3 | 0.72(0.64-0.80) |  | 0.79(0.73-0.86) |
| F2RL3_A_CpG_1 & F2RL3_A_CpG_3 | 0.72(0.64-0.80) |  | 0.79(0.73-0.85) |
| All seven F2RL3_A_CpGs | 0.72(0.65-0.80) |  | 0.80(0.74-0.86) |
| **145 Heart failure cases vs. all 184 controls** | | | |
| **CpG sites** | **AUC, 95% CI ^a^** |  | **AUC, 95% CI ^b^** |
| F2RL3_A_CpG_3 | 0.62(0.55-0.68) |  | 0.69(0.64-0.75) |
| F2RL3_A_CpG_1 & F2RL3_A_CpG_3 | 0.67(0.61-0.73) |  | 0.75(0.69-0.80) |
| All seven F2RL3_A_CpGs | 0.67(0.61-0.73) |  | 0.75(0.69-0.80) |
| **124 NYHA Ⅰ&Ⅱ CHD cases vs. all 184 controls** | | | |
| **CpG sites** | **AUC, 95% CI ^a^** |  | **AUC, 95% CI ^b^** |
| F2RL3_A_CpG_3 | 0.61(0.54-0.67) |  | 0.71(0.66-0.77) |
| F2RL3_A_CpG_1 & F2RL3_A_CpG_3 | 0.67(0.60-0.73) |  | 0.76(0.71-0.82) |
| All seven F2RL3_A_CpGs | 0.69(0.62-0.75) |  | 0.78(0.72-0.83) |

^a^ Logistic regression adjusted for age and gender. ^b^ Logistic regression adjusted for age, gender, smoking, hypertension, TC, LDL, and batch effect.

**Supplementary Table 6.** The methylation of *F2RL3* in CHD patients with variant medical treatments

| **Medicine** | **Group (N)** | **Median of methylation intensity** | | | | | | | | | | |
| --- | --- | --- | --- | --- | --- | --- | --- | --- | --- | --- | --- | --- |
|  |  | **F2RL3_A_CpG_1** | **F2RL3_A_CpG_2/cg03636183** | **F2RL3_A_CpG_3** | **F2RL3_A_CpG_4** | **F2RL3_A_CpG_5** | **F2RL3_A_CpG_6** | **F2RL3_A_CpG_7** | **F2RL3_B_CpG_2** | **F2RL3_B_CpG_4.5** | **F2RL3_B_CpG_6** | **F2RL3_B_CpG_7** |
| ACEI | No (147) | 0.62 | 0.82 | 0.72 | 0.63 | 0.85 | 0.63 | 0.66 | 0.72 | 0.10 | 0.44 | 0.04 |
|  | Yes (33) | 0.58 | 0.82 | 0.64 | 0.61 | 0.83 | 0.61 | 0.62 | 0.69 | 0.10 | 0.43 | 0.04 |
|  | *p-*value * | 0.772 | 0.919 | 0.097 | 0.638 | 0.398 | 0.638 | 0.254 | 0.303 | 0.759 | 0.524 | 0.778 |
| ARB | No (139) | 0.63 | 0.82 | 0.71 | 0.62 | 0.85 | 0.62 | 0.65 | 0.72 | 0.10 | 0.44 | 0.04 |
|  | Yes (41) | 0.62 | 0.82 | 0.71 | 0.65 | 0.85 | 0.65 | 0.66 | 0.69 | 0.09 | 0.44 | 0.04 |
|  | *p-*value * | 0.793 | 0.606 | 0.616 | 0.605 | 0.664 | 0.605 | 0.675 | 0.893 | 0.933 | 0.861 | 0.901 |
| CCB | No (126) | 0.64 | 0.83 | 0.71 | 0.62 | 0.85 | 0.62 | 0.66 | 0.70 | 0.10 | 0.44 | 0.03 |
|  | Yes (54) | 0.61 | 0.82 | 0.70 | 0.64 | 0.84 | 0.64 | 0.66 | 0.73 | 0.09 | 0.42 | 0.04 |
|  | *p-*value * | 0.696 | 0.704 | 0.476 | 0.342 | 0.539 | 0.342 | 0.514 | 0.701 | 0.143 | 0.344 | 0.089 |
| β blocker | No (64) | 0.63 | 0.82 | 0.72 | 0.65 | 0.85 | 0.65 | 0.66 | 0.74 | 0.10 | 0.44 | 0.03 |
|  | Yes (116) | 0.62 | 0.82 | 0.70 | 0.62 | 0.85 | 0.62 | 0.66 | 0.69 | 0.09 | 0.44 | 0.04 |
|  | *p-*value * | 0.881 | 0.775 | 0.166 | 0.307 | 0.894 | 0.307 | 0.664 | **0.032** | 0.874 | 0.919 | 0.478 |
| Spironolactone | No (143) | 0.61 | 0.82 | 0.71 | 0.63 | 0.85 | 0.63 | 0.66 | 0.73 | 0.10 | 0.44 | 0.04 |
|  | Yes (37) | 0.72 | 0.84 | 0.57 | 0.62 | 0.86 | 0.62 | 0.66 | 0.68 | 0.09 | 0.41 | 0.01 |
|  | *p-*value * | 0.532 | 0.413 | 0.192 | 0.555 | 0.401 | 0.555 | 0.829 | **0.040** | 0.090 | 0.790 | **0.026** |
| Digoxin | No (169) | 0.62 | 0.82 | 0.72 | 0.63 | 0.85 | 0.63 | 0.66 | 0.72 | 0.10 | 0.44 | 0.04 |
|  | Yes (11) | 0.59 | 0.79 | 0.49 | 0.61 | 0.74 | 0.61 | 0.61 | 0.68 | 0.05 | 0.41 | 0.01 |
|  | *p-*value * | 0.603 | 0.745 | **0.001** | 0.147 | **0.010** | 0.147 | 0.132 | 0.463 | **0.007** | 0.661 | 0.136 |
| Nitrates | No (84) | 0.65 | 0.83 | 0.72 | 0.62 | 0.85 | 0.62 | 0.65 | 0.73 | 0.10 | 0.42 | 0.04 |
|  | Yes (96) | 0.60 | 0.82 | 0.70 | 0.63 | 0.85 | 0.63 | 0.66 | 0.69 | 0.09 | 0.45 | 0.04 |
|  | *p-*value * | 0.341 | 0.689 | 0.470 | 0.147 | 0.907 | 0.147 | 0.327 | 0.424 | 0.708 | 0.127 | 0.738 |
| Aspirin | No (42) | 0.65 | 0.82 | 0.72 | 0.62 | 0.86 | 0.62 | 0.66 | 0.69 | 0.09 | 0.45 | 0.03 |
|  | Yes (138) | 0.61 | 0.83 | 0.70 | 0.63 | 0.85 | 0.63 | 0.66 | 0.73 | 0.10 | 0.44 | 0.04 |
|  | *p-*value * | 0.899 | 0.635 | 0.321 | 0.583 | 0.420 | 0.583 | 0.539 | 0.219 | 0.838 | 0.229 | 0.520 |
| Clopidogrel | No (79) | 0.61 | 0.81 | 0.71 | 0.62 | 0.85 | 0.62 | 0.66 | 0.73 | 0.09 | 0.44 | 0.03 |
|  | Yes (101) | 0.67 | 0.84 | 0.70 | 0.63 | 0.85 | 0.63 | 0.66 | 0.69 | 0.10 | 0.44 | 0.04 |
|  | *p-*value * | 0.534 | 0.195 | 0.652 | 0.681 | 0.508 | 0.681 | 0.802 | 0.533 | 0.828 | 0.926 | 0.353 |
| Warfarin | No (175) | 0.62 | 0.82 | 0.71 | 0.63 | 0.85 | 0.63 | 0.66 | 0.71 | 0.10 | 0.44 | 0.04 |
|  | Yes (5) | 0.58 | 0.85 | 0.73 | 0.58 | 0.91 | 0.58 | 0.74 | 0.68 | 0.05 | 0.47 | 0.02 |
|  | *p-*value * | 0.947 | 0.512 | 0.961 | 0.743 | 0.118 | 0.743 | 0.481 | 0.671 | 0.325 | 0.839 | 0.796 |
| Statin | No (19) | 0.70 | 0.84 | 0.71 | 0.62 | 0.85 | 0.62 | 0.67 | 0.71 | 0.09 | 0.45 | 0.02 |
|  | Yes (161) | 0.61 | 0.82 | 0.71 | 0.63 | 0.85 | 0.63 | 0.66 | 0.71 | 0.10 | 0.44 | 0.04 |
|  | *p-*value * | 0.800 | 0.492 | 0.612 | 0.683 | 0.229 | 0.683 | 0.478 | 0.410 | 0.156 | 0.698 | 0.394 |
| Antacids | No (119) | 0.65 | 0.83 | 0.70 | 0.62 | 0.85 | 0.62 | 0.66 | 0.71 | 0.09 | 0.43 | 0.04 |
|  | Yes (61) | 0.60 | 0.82 | 0.72 | 0.63 | 0.85 | 0.63 | 0.65 | 0.71 | 0.10 | 0.46 | 0.03 |
|  | *p-*value * | 0.159 | 0.919 | 0.770 | 0.822 | 0.806 | 0.822 | 0.911 | 0.403 | 0.911 | 0.149 | 0.761 |

* The *p*-values were calculated by the Mann-Whitney test, and significant *p*-values are in bold. ACEI, Angiotensin converting enzyme inhibitor; ARB, Angiotensin receptor blocker; CCB, Calcium channel blockers.
