# Supplementary material for: F2RL3 Methylation in the Peripheral Blood as a Potential Marker for the Detection of Coronary Heart Disease: A Case-Control Study
Source: Front Genet. 2022 Mar 24;13:833923. doi: 10.3389/fgene.2022.833923 (PMC8996303; doi:10.3389/fgene.2022.833923)
Supplement: Supplementary file 2 [file DataSheet2.doc]

**SUPPLEMENTAL MATERIAL**

**Title:** *F2RL3* methylation in the peripheral blood as a potential marker for the detection of [coronary heart disease](http://www.baidu.com/link?url=BjTnQxOUyMa23gY-kV8Amz0OQ3GmdDJbQnWekDeUCsUCRyxBnNhCsmkGz_nN3phTzZ-g0ixpTiHihJ4wHCXSjZqaiqXAU1B0hIwNFasp69U5JSDoQxV9WKwWdcqUMHOM): a case-control study

**Running Title:** DNA Methylation in CHD

**Xiaojing Zhao^1,2†^, Liya Zhu^3†^, Qiming Yin^3^, Zhenguo Xu^4,5^, Qian Jia^1,2^,** [**Rongxi Yang**](http://loop.frontiersin.org/people/974610/overview)**^3*^, Kunlun He^1,2*^**

^1^Military translational medicine lab, Medical Innovation Research Division, Chinese PLA General Hospital, Beijing, China

^2^Beijing Key Laboratory of Chronic Heart Failure Precision Medicine, Medical Innovation Research Division, Chinese PLA General Hospital, Beijing, China

^3^Department of Epidemiology and Biostatistics, School of Public Health, Nanjing Medical University, Nanjing, China

^4^The First Medical Center, Chinese PLA General Hospital, Beijing, China

^5^The medical school of Chinese PLA, Beijing, China

*** Correspondence:**

Rongxi Yang

[rongxiyang@njmu.edu.cn](mailto:rongxiyang@njmu.edu.cn)

Kunlun He

[kunlunhe@plagh.org](mailto:kunlunhe@plagh.org)

^†^These authors have contributed equally to this work and share first authorship

**Supplementary Figures**

**A**


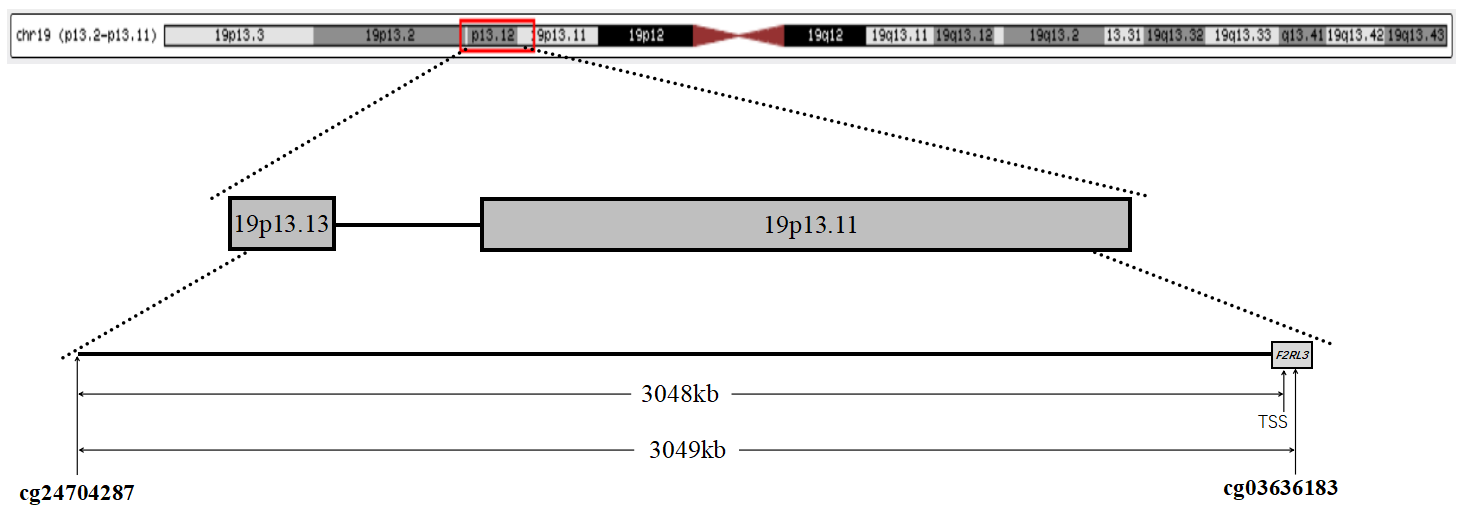


**B**


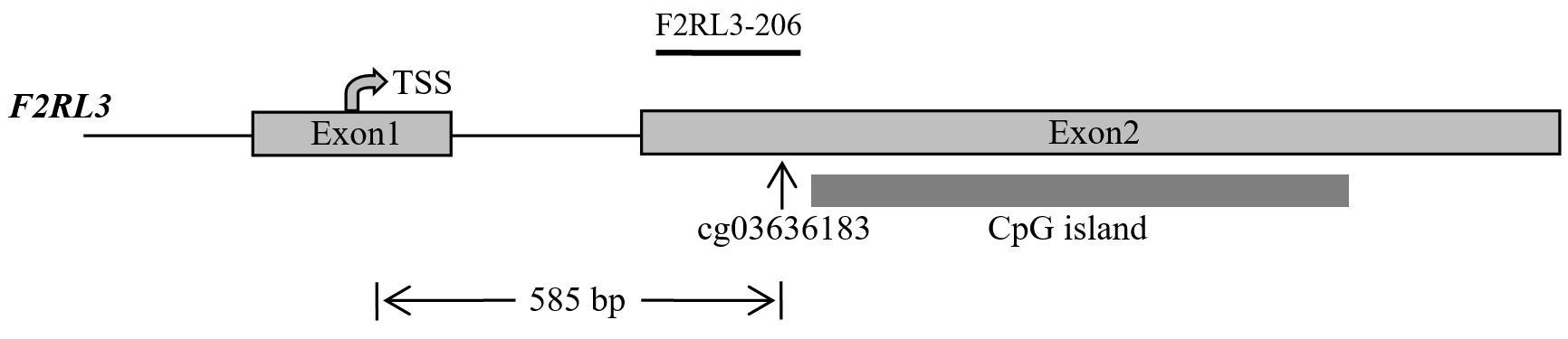


**C**

GGTTCATCAGCAGCATGGTGGAGGGCAGC**CG**AGGTGCCTG**CG**TGGCCAGCACCCACAG**CG**CCAGCCCATTGGC**CG**GCAGCCCCACCACCAGGACCAGCCCATAGAGGG**CG**GGCACCAGCCTGGTGGGCACCCAGCCCAGAAGCAGTGCC**CG**TGAGCTGTC**CG**GGAGCTCCAGGGTGTCACTGTCATTGGCACAGACTTGGCCTGGG

**D**

GTCACTGAGCTGTGGCCCTGGGCAGAGCAGGGCTGGGTGGTCTAGGATGTGGGGCCTCGGTGCCTGGGGAACTGACTT**CG**AAGATGCCAAGCCCCCTGGCCTCCCGCCTGGCCTAGGTTCTGGGGTCACCCTCCAGGACTGTGGTGGCCCACACCCTGAGAATGCCTTGGAGAGCCTAGAGGCCCTGAGCCTTGGGCCTGGGAAT**CG**GTG**CG**TTGAGGGAGGTGGATAGGG**CG**CAGACACCAGTGACTGCATTCAAGGCCAGGGGACCCCCACAGCCAGGCCCTCACTGCAAAATGTACCTATGTGGGGAGAAGGCAGGGGTGGACCTGATGA**CG**GGGTGTACCACTCTACTAAGGGTTCCCAACATGAAGGTGAGG

**Supplementary Figure 1.** Schematic diagram and the sequence of *F2RL3* amplicons. **(A)** A schematic diagram of the location of cg03636183 and cg24704287 (from the UCSC Genome Browser). cg03636183 is located at the body of *F2RL3***,** cg24704287 is located at 3048 kb upstream of *F2RL3*. **(B)** The location of the F2RL3_A amplicon in *F2RL3*. The F2RL3_A amplicon is located at the second exon of *F2RL3* and covers the main hit CpG cg03636183. The 585bp between the transcript start site (TSS) and cg03636183 has been labelled. **(C)** The sequence of the F2RL3_A amplicon examined by the EpiTyper assay (chr19:17,000,421-17,000,626, build 37/hg19, defined by the UCSC Genome Browser). The EpiTyper assay determined the methylation levels of 7 CpGs in this amplicon and yielded 7 distinguishable mass peaks. The CpG sites that could be measured are in bold, cg03636183 is in bold and underlined. **(D)** The sequence of the F2RL3_B amplicon examined by the EpiTyper assay (chr19:13,951,024-13,951,400, build 37/hg19, defined by the UCSC Genome Browser). The EpiTyper assay determined the methylation levels of 5 CpGs in this amplicon and yielded 4 distinguishable mass peaks. The CpG sites that could be measured are in bold.

**
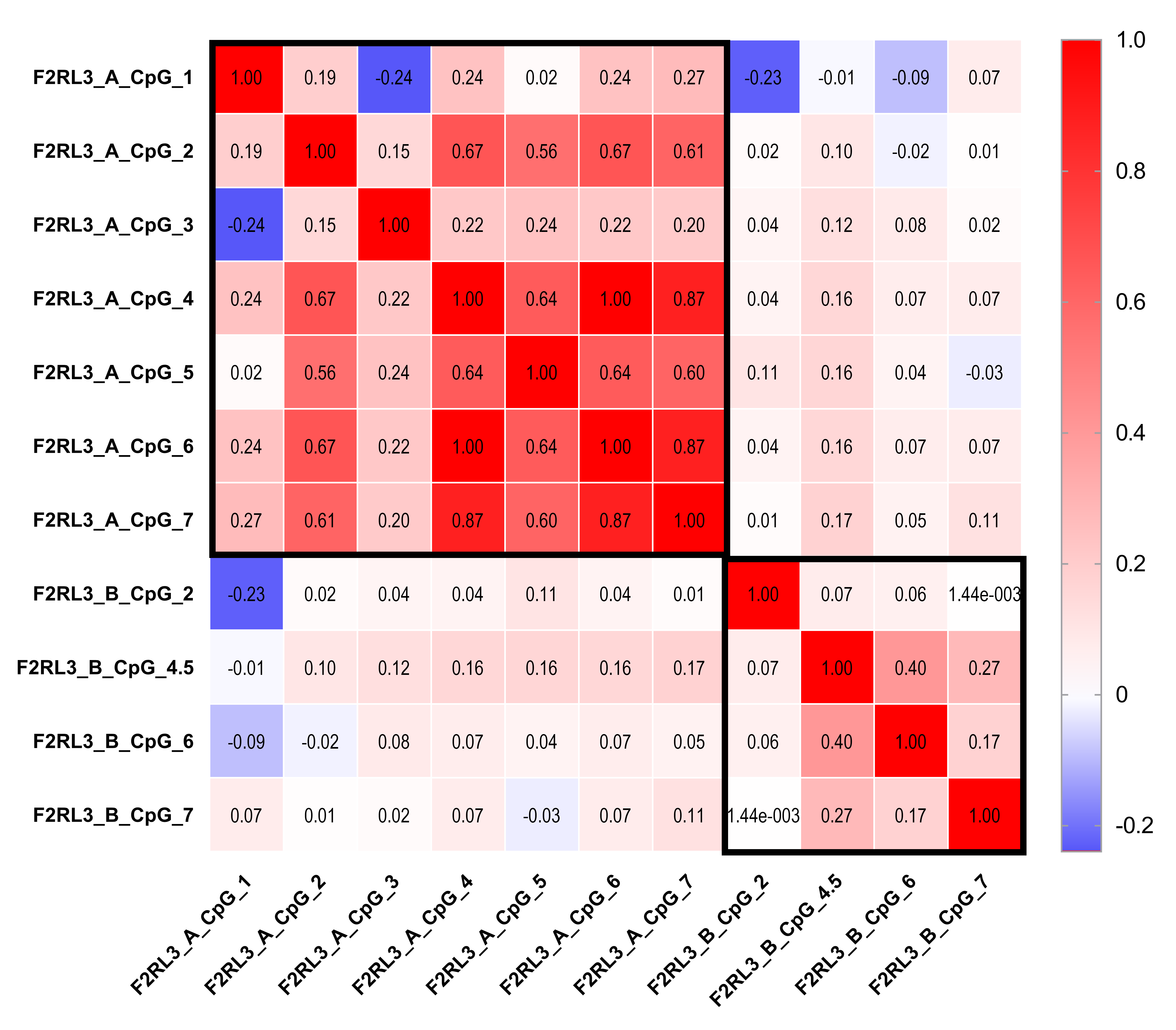
**

**Supplementary Figure 2**. A heatmap of the correlation among the CpG sites in the F2RL3_A amplicon and F2RL3_B amplicon. The positively correlated CpG sites are in red, and the negatively correlated CpG sites are in blue. The color bar represents the strength of correlation as Spearman Rho. The CpG sites in the F2RL3_A amplicon and F2RL3_B amplicon are framed respectively.
